# Chronic obstructive pulmonary disease and risk of digestive diseases: observational and mendelian randomization analyses in the China Kadoorie Biobank

**DOI:** 10.1186/s12931-026-03825-w

**Published:** 2026-07-17

**Authors:** Yuxuan Zhao, Yalei Ke, Dianjianyi Sun, Pei Pei, Ling Yang, Iona Y. Millwood, Robin G. Walters, Yiping Chen, Huaidong Du, Junshi Chen, Zhengming Chen, Jun Lv, Liming Li, Canqing Yu, the China Kadoorie Biobank Collaborative Group, Robert Clarke, Rory Collins, Richard Peto, Daniel Avery, Maxim Barnard, Derrick Bennett, Ruth Boxall, Zhengming Chen, Johnathan Clarke, Ahmed Edris Mohamed, Hannah Fry, Prapthi Harish, Pek Kei Im, Andri Iona, Christiana Kartsonaki, Kshitij Kolhe, Hubert Lam, Kuang Lin, James Liu, Sam Morris, Qunhua Nie, Alfred Pozarickij, Maryanm Rahmati, Paul Ryder, Maruf Sarder, Dan Schmidt, Becky Stevens, Iain Turnbull, Baihan Wang, Lin Wang, Neil Wright, Xiaoming Yang, Pang Yao, Dianjianyi Sun, Yuanjie Pang, Yuting Han, Can Hou, Qingmei Xia, Chao Liu, Lang Pan, Xiao Han, Honglu Bian, Xinxin Chen, Zengchang Pang, Ruqin Gao, Kunzheng Lv, Shanpeng Li, Haiping Duan, Shaojie Wang, Yongmei Liu, Ranran Du, Liang Cheng, Xiaocao Tian, Hua Zhang, Dan Hu, Xiaoyan Zheng, Yujie Wan, Wei Sun, Shichun Yan, Yong Zhou, Chi Wang, Zhenyuan Wu, Lishun Zhai, Zhaoxi Pang, Shiwen Dong, Li Liu, Dapeng Yin, Bin He, Ying Liu, Xingren Wang, Tingting Ou, Xiangyang Zheng, Dewei Zheng, Shuai Yang, Lihui Li, Xingjiao Chen, Yan Xu, Jinyi Zhou, Ran Tao, Jian Su, Xikang Fan, Xuejia Chen, Yuxiao Huang, Yan Lu, Yujie Hua, Li Xing, Shuxian Wang, Jianrong Jin, Juping Ma, Kaifei Zhu, Hongfu Ren, Xingfeng Shen, Ge Zhong, Wei Mao, Zhenzhen Lu, Yanxu Zhong, Lifang Zhou, Rong Pan, Jian Lan, Xiaoping Tan, Jinxue Tan, Yishan Xie, Liuping Wei, Liyuan Zhou, Sisi Wang, Xianping Wu, Ningmei Zhang, Xiaofang Chen, Xiaoyu Chang, Zhuo Wang, Yujin He, Mingqiang Yuan, Ling Wang, Zhaodong Wang, Qiang Sun, Yang Lin, Faqing Chen, Xiaolan Ren, Lijun Chang, Feiming Zhong, Jianjun Feng, Weijie Hu, Xiaofang Zhang, Yalin Chen, Honghong Wang, Jun Wang, Linqi Diao, Zhiwei Han, Dengjun Zhu, Kai Kang, Shixian Feng, Wenjie Yang, Huizi Tian, Yali Yan, Bing Han, Li Gao, Shaofang Li, Tianfang Xing, Wei Tang, Xiaolin Li, Huarong Sun, Xiaocong Zhao, Chen Hu, Pan He, Xukui Zhang, Jin Yuanyuan, Lan Luo, Min Yu, Ruying Hu, Hao Wang, Weiwei Gong, Jieming Zhong, Meng Wang, Chunxiao Xu, Keqing Gong, Hao Xu, Yuan Cao, Kaixu Xie, Lingli Chen, Xiaomei Tu, Junlong Pan, Xiaojun Li, Li Yin, Huilin Liu, Yuan Liu, Lei Yin, Xian Xie, Jing Wang, Bo Xiao, Zongwei Deng, Yuan Peng, Libo Zhang, Chan Qu, Li Deng, Qili Jiang, Yanling Chen, Junshi Chen, Zhengming Chen, Liming Li, Jun Lv, Robin Walters, Yiping Chen, Huaidong Du, Iona Millwood, Ling Yang, Pei Pei, Canqing Yu, Ying Li

**Affiliations:** 1 Department of Epidemiology and Biostatistics, School of Public Health, Peking University Health Science Center, Beijing, 100191 China; 2 Peking University Center for Public Health and Epidemic Preparedness & Response, Beijing, 100191 China; 3 Key Laboratory of Epidemiology of Major Diseases (Peking University), Ministry of Education, Beijing, 100191 China; 4 Clinical Trial Service Unit & Epidemiological Studies Unit (CTSU), Nuffield Department of Population Health, University of Oxford, Oxford, OX3 7LF UK; 5 China National Center for Food Safety Risk Assessment, Beijing, 100022 China; 6 State Key Laboratory of Vascular Homeostasis and Remodeling, Peking University, Beijing, 100191 China

**Keywords:** Chronic obstructive pulmonary disease, Digestive diseases, Preserved ratio impaired spirometry, Mendelian randomization

## Abstract

**Background:**

Several digestive diseases have been reported as comorbidities of COPD. However, consistent evidence supporting their associations is limited. Our study aims to investigate the associations between prevalent COPD and incident digestive diseases in the Chinese population.

**Methods:**

We used data from the China Kadoorie Biobank (CKB), enrolling 512,724 participants aged 30–79 years at baseline from 10 areas in China. COPD was defined by spirometry (without bronchodilator) and self-reported emphysema/chronic bronchitis at baseline. We employed stratified Cox proportional regression models to assess the prospective associations of prevalent COPD and airflow obstruction severity with the risks of incident digestive diseases. A total of 31 digestive diseases were included, including the overall digestive diseases, 15 major disease categories, and 15 specific subtypes nested within the major categories. We further conducted one-sample Mendelian randomization analyses to assess the associations of genetically predicted COPD with digestive diseases.

**Results:**

During a median follow-up of 15.3 years, a total of 131,993 participants experienced at least one hospitalization or death due to the included digestive diseases. For the composite outcome and major categories, prevalent COPD at baseline was significantly associated with a higher risk of overall digestive diseases (hazard ratio and 95% confidence interval: 1.05 [1.03–1.08]), gastrointestinal inflammation (1.11 [1.07–1.15]) and abdominal pain (1.20 [1.12–1.28]). Dose–response associations of airflow obstruction severity with the overall digestive diseases (*P* value for trend: < 0.001) and six other disease types were identified. In MR analyses, a genetically predicted higher probability of COPD was significantly associated with overall digestive diseases (OR per doubling of genetically-predicted COPD prevalence: 3.21 [1.71–6.03]) and three other disease types.

**Conclusions:**

Prevalent COPD is associated with various digestive diseases in the Chinese population, with MR analyses further supporting a potential causal link. These findings underscore the importance of considering disease severity when managing COPD-related digestive diseases. While COPD was defined based on baseline spirometry in this study, future research incorporating post-bronchodilator assessment could further refine these associations and help distinguish COPD from other reversible airflow limitations.

**Supplementary Information:**

The online version contains supplementary material available at https://doi.org/10.1186/s12931-026-03825-w.

## Introduction

Chronic obstructive pulmonary disease (COPD) is one of the most commonly seen respiratory diseases worldwide, characterized by airflow obstruction and irreversible decline in lung function [1]. It is also a leading contributor to the global disease burden and economic burden, resulting in 3.28 million deaths and 74.43 million Disability-Adjusted Life Years, according to the Global Burden of Disease study, with China accounting for the most significant financial burden of COPD [2–4].

COPD usually coexists with present or subsequent comorbidities [5], making it more complex for disease management. Among the wide range of health conditions, COPD and digestive diseases have been accumulating attention, given the increasing interest in the ‘lung-gut’ axis [6]. Previous studies have observed higher prevalence or increased risks for several digestive diseases or symptoms among COPD patients [6–10]. However, many of them were based on case–control designs, prospective associations and consistent causal relationships were less inferred [11]. Besides, as a progressive disease, the different severity stages of COPD or airflow obstruction might have varying impacts on the digestive system, but little is known about it. Although many digestive diseases alone were not life-threatening, they can still cause severe complications and even fatal outcomes for patients with complications of COPD [12, 13]. Therefore, it is still of significant implications for public health to find and validate their relationships for prevention and management purposes.

In our study, we aim to systematically assess the associations between COPD and various digestive diseases based on prospective analyses, and provide evidence for potential causal associations through MR analyses in the China Kadoorie Biobank (CKB), comprising 0.5 million adults.

## Methods

### Study design and participants

Details of the study design and survey methods have been described previously [14]. In brief, we used data from the CKB, a large-scale prospective cohort comprising 512,724 participants aged 30 to 79 years from five urban (Qingdao, Harbin, Liuzhou, Suzhou, and Haikou; represented by the city name) and five rural (Sichuan, Zhejiang, Hunan, Gansu, and Henan; represented by the provincial name) areas across China, collected at baseline from 2004 to 2008. The study areas were chosen according to local disease patterns, exposure to certain risk factors, population stability, quality of death and disease registries, local commitment and capacity. Door-to-door invitations letters were delivered to potentially eligible participants within administrative units (rural villages or urban residential committees), and they were invited to the local assessment centers for further questionnaire survey, physical examinations, and biological sample collection, all of which were guided by trained staff. After that, every four-to-five years, 5% of the research subjects would be selected for resurveys.

Overall, the number recruited from each area ranged from about 30,000 in Haikou to over 63,000 in Henan. The detailed description of the population characteristics was shown in Supplementary Table 1. Of the 512,724 participants, 59.0% were female, 56% were from rural areas, and the mean age was 52.0 years. Nearly all were married, and about half of the participants had at least 6 years of formal education. The prevalence rates for current smokers and current daily drinkers were 29.4% and 9.0%, respectively, and were both much higher in males than females.

**Fig. 1:**
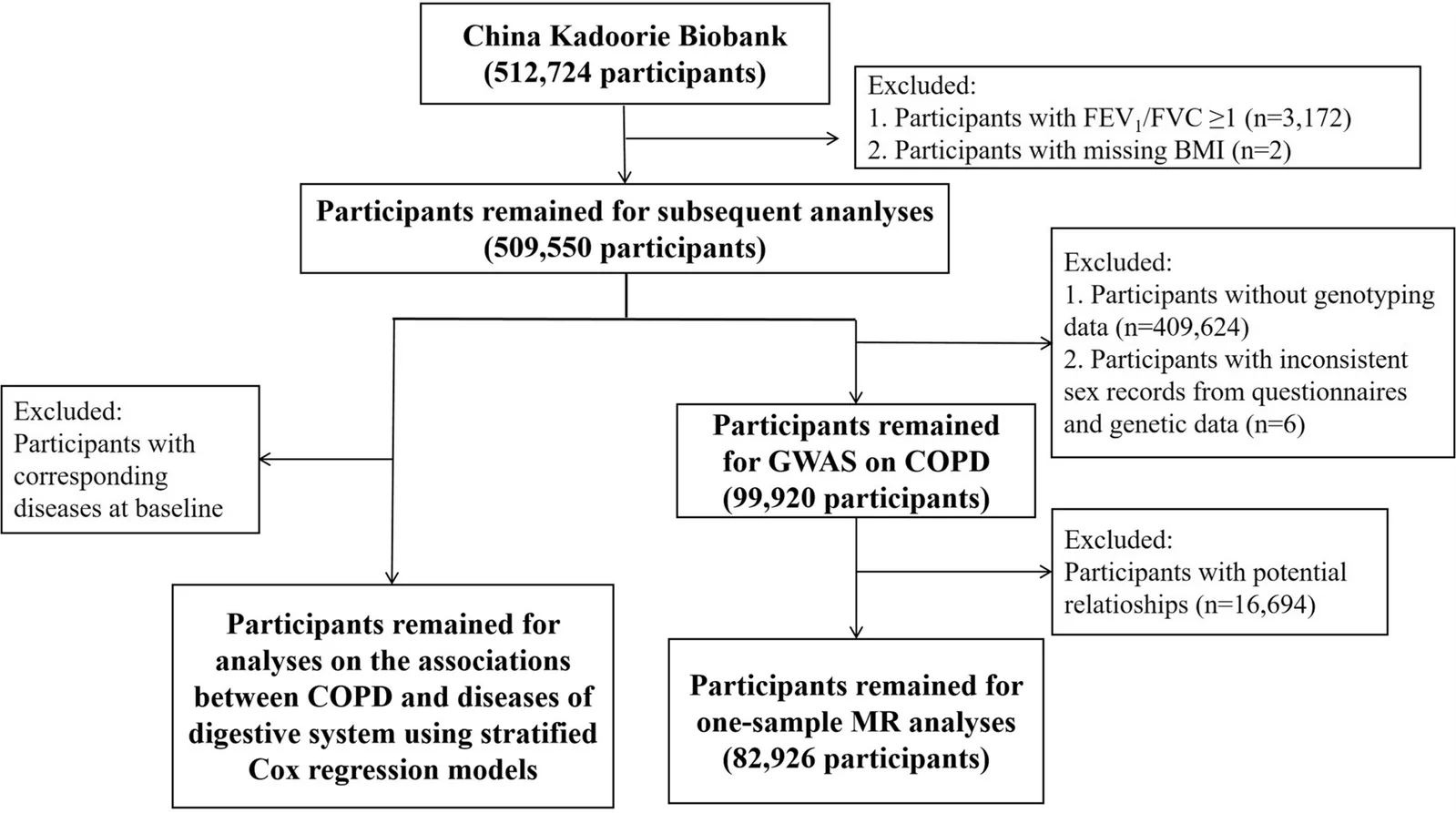
The flowchart of the inclusion of the study population. Abbreviations: COPD, chronic obstructive pulmonary disease; GWAS, genome-wide association study; FEV_1_, forced expiratory volume in one second; FVC, forced vital capacity; BMI, body mass index; MR, Mendelian randomization

We excluded participants with an implausible lung spirometry (i.e., the ratio of the largest Forced Expiratory Volume in the 1 st second (FEV_1_) and largest Forced Vital Capacity(FVC) in two successful measure ≥ 1.0, n = 3,172) or missing data for covariates (body mass index [BMI], n = 2), leaving 509,550 participants for the present analysis. Detailed descriptions of the inclusion criteria of the study population are shown in Fig. 1.

### Spirometry and identification of COPD

Details of the spirometry at baseline have been previously reported [15]. FEV_1_ and FVC were measured using a handheld micro-spirometer by trained technicians according to recommended procedures, and two successful maneuvers were recorded. The larger of the two measurements for each FEV_1_ and FVC was used to calculate the FEV_1_/FVC ratio and for further analysis. Bronchodilators were not applied at baseline, and the spirometry defined cases can only be interpreted as “pre-bronchodilator COPD”. Predicted values for FEV_1_ and FVC were calculated based on the Global Lung Function Initiative 2012 equations for the Southeast and Northeast Asian populations [16] COPD was defined based on either the Global Initiative for Obstructive Lung Disease (GOLD) criterion (FEV_1_/FVC < 0.7) [1], or self-reported emphysema/chronic bronchitis at baseline. The severity of airflow obstruction in COPD patients was graded according to the GOLD criteria (GOLD stage 1, mild: FEV_1_ ≥ 80% predicted; GOLD stage 2, moderate: 50% ≤ FEV_1_ < 80% predicted; GOLD stage 3, severe: 30% ≤ FEV_1_ < 50% predicted; and GOLD stage 4, very severe: FEV_1_ < 30% predicted). Preserved ratio with impaired spirometry (PRISm) was defined by FEV_1_ < 80% predicted among non-COPD individuals [17].

### Assessment of covariates

All participants underwent interviewer-administered laptop-based questionnaires at baseline, which covered sociodemographic characteristics, lifestyle factors, and medical history. Subsequently, they undertook physical measurements. The total daily physical activity level was calculated by multiplying the metabolic equivalent of tasks (MET) value assigned to a specific type of physical activity by the daily hours spent on that activity, then summing the resulting MET hours per day (MET‐h/d) for all activities. Body weight, height, and blood pressure were measured by trained staff using a standard protocol and calibrated instruments. Prevalent hypertension was defined as systolic blood pressure ≥ 140 mmHg, diastolic blood pressure ≥ 90 mmHg, self-reported physician-diagnosed hypertension, or self-reported use of antihypertensive drugs. All participants provided a 10 mL non-fasting blood sample for an immediate on-site test of random plasma glucose. Diabetes was defined as fasting blood glucose ≥ 7.0 mmol/L, random blood glucose ≥ 11.1 mmol/L, or self-reported physician-diagnosed diabetes.

### Follow-up and ascertainment of outcomes

CKB adopted several ways to collect disease events and mortality status. All 10 study regions were under China's Disease Surveillance Points system and Major Chronic Disease Surveillance system at baseline and are linked to the national health insurance claim system to collect each participant's hospitalization episodes. The follow-up has been ongoing from baseline to the present, with the most recent available outcome data censored as of December 31, 2022. All events recorded were standardized to the International Classification of Diseases, 10th Revision (ICD-10). To capture more clinically meaningful concepts for our research, the ICD-10 codes were mapped to the phecodeX map in the following analyses [18].

The included outcomes of the digestive system and the coding rules for mapping from ICD-10 codes to phecodeX codes were presented in Supplementary Table 2. Based on the selection criteria of at least 100 unique records for each outcome and prior knowledge of the potential COPD-related diseases, we included a total of 31 disease types in our analyses. Only the record of the first occurrence was used for participants with multiple records of the ***s***ame condition.

### Statistical analyses

#### Observational analyses

Means and percentages of baseline characteristics were calculated for prevalent COPD at baseline using linear regression for continuous variables and logistic regression for categorical variables, adjusted for age, sex, and study area when appropriate. Incident rates per 1,000 person-years were calculated for individuals with and without COPD (Supplementary Table 2).

Firstly, we used stratified Cox proportional hazards regression to estimate hazard ratios (HRs) with 95% confidence intervals (CIs), comparing risks in COPD patients versus non-COPD participants. All analyses were stratified by age-at-risk (in five-year groups, ranging from 30 to 100), sex, and 10 study areas, where appropriate, and adjusted for education, household income, occupation, marital status, alcohol consumption, smoking status, passive smoking status, physical activity levels, primary cooking fuel, primary heating fuel usage, and consumption frequency of fresh fruits, fresh vegetables, meat, general obesity and abdominal obesity, as well as self-rated health status, prevalent hypertension and prevalent diabetes at baseline. Detailed grouping information of the covariates has been listed in Supplementary Table 3. The assumption of proportional hazards was verified using the Schoenfeld residuals. Participants with known digestive diseases relevant to the targeted outcomes at baseline were excluded from analyses (Supplementary Table 2). Censoring was defined by death, loss to follow-up, or global censoring by 31 December 2022. We additionally summarised the number of occurrence for each digestive diseases and assessed the associations between COPD and the frequency of occurrence through negative binomial regressions.

Next, we conducted subgroup analyses by several factors: age, sex, BMI, smoking status, and drinking status. *P-values* for interactions of COPD with these factors were obtained from likelihood-ratio tests comparing the two models before and after adding the interaction term.

We further explored the associations between airflow obstruction severity and the incidence of diverse digestive disease categories. The HRs for each severity group were calculated and compared to those of non-COPD participants with FEV_1_% predicted values of 80% or higher, adjusting for the same covariates mentioned above. *P* values for trend were calculated by treating the multiple categorical variables representing airflow obstruction severity as continuous variables in the regression models. Since smoking has been regarded as one of the most significant shared risk factors for COPD and digestive diseases, we also conducted subgroup analyses by smoking status on the associations between airflow obstruction severity and the incidence of diverse digestive disease categories.

For sensitivity analyses, first, we excluded participants whose follow-up time was less than two years. Second, considering anxiety could be a potential confounding factor between prevalent COPD and the incidence of digestive diseases, we included anxiety, measured by self-reported continuous anxiety, continuous pain, panic attacks, and phobic symptoms reported at baseline, as an additional covariate in the regression models. Third, to minimize the misclassification of asthma due to the lack of post-bronchodilator measurements, we excluded participants with self-reported asthma (n = 2,805) at baseline. We also conducted subgroup analyses by smoking status among participants without self-reported asthma, as smoking could influence bronchodilator response. Fourth, we excluded participants who have reported the history of aspirin usage at baseline. Fifth, as obesity, hypertension, and diabetes may potentially serve as mediating factors between COPD and digestive diseases, we excluded these factors from the covariates. Sixth, Fine-Gray competing risk models were applied to account for death as a competing event for digestive disease hospitalization.

#### Mendelian randomization (MR) analyses

The selection of instrumental variables and the construction of genetic risk score (GRS) among CKB participants were shown in Supplementary Text 1, Supplementary Fig. 1 and Supplementary Table 4. Briefly, we conducted genome-wide association studies (GWAS) on COPD in the CKB population. We selected independent lead SNPs with a significant level below 5 × 10^–8^ and good quality to construct the unweighted GRS as the instrumental variable(IV). To satisfy the assumptions of MR, we excluded SNPs associated with the covariates adjusted (Supplementary Table 6 and 7). Finally, 2 SNPs remained to construct the GRS named ‘CKB GRS 5e8’ and the F-statistics was 42.9.

Two-stage regression analyses were applied for one-sample MR analyses. We fitted logistic regressions in the first stage to obtain predicted values of COPD, adjusting for the top ten principal components and genetic arrays. Stratified Cox proportional hazards regression models were then applied to obtain the effects of genetically predicted COPD on digestive diseases, adjusting for covariates same as those in the observational studies, as well as the top ten principal components and genetic arrays. To account for relatedness, we randomly excluded one participant from a pair of related participants with a relationship coefficient greater than 0.088, leaving 82,926 participants in the two-stage analyses. The outcomes included in MR analyses were diseases found to be associated with COPD or airflow obstruction severity.

For sensitivity analyses, we conducted a meta-GWAS using the summary statistics from both CKB and Biobank Japan through the METAL software [19, 20]. The description and summary results for the GWAS in Biobank Japan are available elsewhere [20]. The procedures for obtaining IVs and completing the MR analyses were identical to those in the primary analyses. Results for meta-GWAS and information on the clumped SNPs are also shown in Supplementary Fig. 1 and Supplementary Table 4. Five SNPs were left for the construction of GRS named ‘Meta GRS 5e8’ and the F-statistics was 21.1. To try to reduce the overestimation of the effects introduced by selecting IVs from the same cohort, a sensitivity analysis using IVs derived only from the independent BBJ GWAS (namely ‘BBJ GRS 5e8’) was conducted. The IVs and selection procedure from BBJ GWAS were supplied in Supplementary Text 1, Supplementary Table 5–7. However, since the initial BBJ-derived GRS had a low F-statistic (4.7) due to two SNPs that showed opposite directions of association with COPD in CKB versus BBJ, we constructed another GRS excluding these heterogeneous SNPs (namely ‘BBJ GRS 5e8 nohet’) and the F-statistic increased to 17. We also conducted a sensitivity analysis excluding obesity, hypertension, and diabetes from the covariates for the reasons stated above.

Unless otherwise stated, all statistical analyses were performed using R (version 4.3.3). The Benjamini–Hochberg procedure was used to adjust *P-values* across 31 diseases. Associations were reported based on their nominal significance (*P* < 0.05) and study-wide significance (False Discovery Rate, *FDR* < 0.05).

## Results

### Baseline characteristics

Baseline characteristics of the included participants were presented in Table 1. The mean (SD) age was 52.0 (10.7) at baseline, with 59.1% women and 43.8% residing in urban areas. A total of 37,032 (7.3%) participants were defined as COPD patients at baseline, and they were more likely to be older, males, live in rural areas, earn less income, be current smokers, eat less meat, have lower BMI and waistline, and rate themselves as poor health.

**Table 1:** Characteristics of study participants by prevalent COPD at baseline

	Overall	Non-COPD participants	COPD patients
N	509,550	472,518 (92.7)	37,032 (7.3)
Age (mean [SD])	52.0 (10.7)	51.5 (10.4)	58.6 (10.5)
Female (%)	301,035 (59.1)	282,440 (59.7)	18,595 (51.8)
Urban areas (%)	223,368 (43.8)	209,500 (44.5)	13,868 (35.4)
Education (%)			
Primary school or below	259,463 (50.9)	234,159 (50.7)	25,304 (53.4)
Middle or high school	220,395 (43.3)	210,099 (43.5)	10,296 (39.7)
Technical school/college or above	29,692 (5.8)	28,260 (5.8)	1432 (6.2)
Occupation (%)			
Retired or unemployed	159,117 (31.2)	145,458 (31.1)	13,659 (32.2)
Agriculture or factory-related	284,813 (55.9)	264,366 (55.9)	20,447 (55.5)
Others	65,620 (12.9)	62,694 (12.9)	2,926 (12.3)
Income (%)			
< 10,000 CNY/year	144,226 (28.3)	129,394 (27.9)	14,832 (32.7)
10,000–19,999 CNY/year	147,959 (29.0)	138,176 (29.1)	9,783 (28.3)
≥ 20,000 CNY/year	217,365 (42.7)	204,948 (43.0)	12,417 (37.7)
Married (%)	461,532 (90.6)	429,858 (90.7)	31,674 (89.4)
Current smokers (%)	149,602 (29.4)	134,655 (29.1)	14,947 (32.4)
Daily drinkers (%)	45,666 (9.0)	41,305 (9.1)	4,361(8.0)
MET-h/d (mean [SD])	21.09 (13.9)	21.14 (11.9)	20.46 (12.21)
Daily intake of fresh fruits (%)	95,396 (18.7)	90,140 (18.7)	5,256 (18.3)
Daily intake of fresh vegetables (%)	482,904 (94.8)	447,573 (94.8)	35,331 (94.5)
> 4 days/week intake of meat (%)	239,521 (47.0)	224,957 (47.2)	14,564 (44.8)
Ever passive smokers (%)	306,394 (60.1)	284,716 (60.2)	21,678 (59.7)
Exposure to cook pollution (%)	315,255 (61.9)	289,499 (61.8)	25,756 (63.0)
Exposure to heat pollution (%)	403,794 (79.3)	373,258 (79.2)	30,536 (79.1)
BMI ≥ 24kg/m^2^ (%)	222,644 (43.7)	210,097 (44.4)	12,547 (34.8)
Abdominal obesity (%)	123,656 (24.3)	116,300 (24.7)	7,356 (19.4)
Self-rated poor health (%)	52,864 (10.4)	45,128 (9.7)	7,736 (17.2)
Hypertension (%)	179,526 (35.2)	164,147 (35.5)	15,379 (32.2)
Diabetes (%)	30,100 (5.9)	27,814 (6.0)	2,286 (5.0)

Values are means or percentages of participants adjusted for age, sex, and study areas when appropriate

All *P for difference* < 0.001, except for diabetes (*P* = 0.025)

*Abbreviations*: *COPD* chronic obstructive pulmonary disease, *SD* standard deviation, MET-h/d metabolic equivalents of task per hour per day, *BMI* body mass index

### Associations of COPD at baseline with selected digestive diseases

During a median follow-up of 15.3 years (IQR: 10.1–16.6), 131,993 participants experienced at least one hospitalization or death due to the included digestive system diseases. Only events that occurred for the first time were analyzed, and the number of events for different disease types ranged from 115 to 47,585 (Supplementary Table 2).

**Fig. 2:**
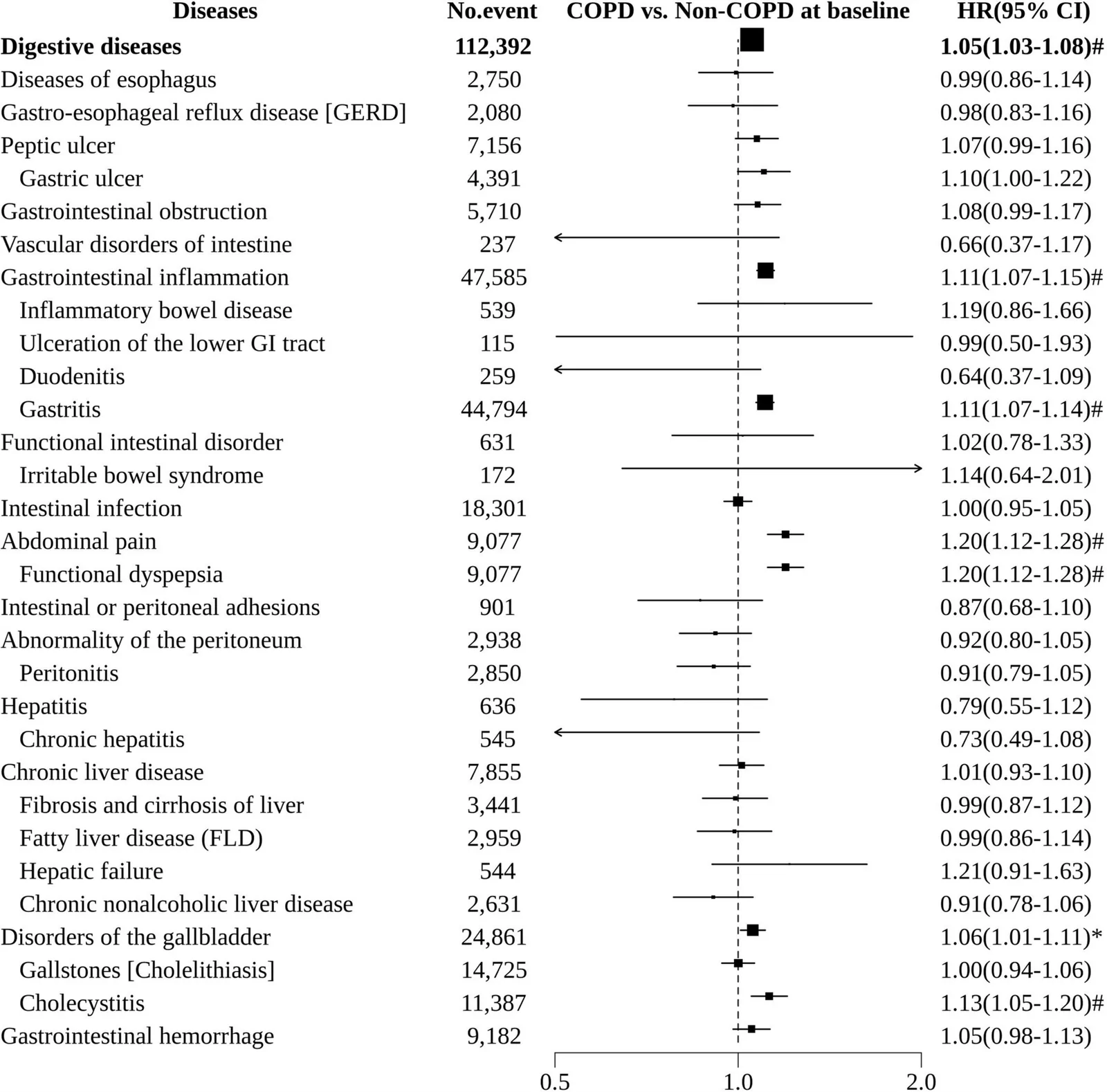
Associations of prevalent COPD at baseline with selected digestive diseases. The centers of the squares represent the point estimates for HRs, and the edges represent the lower and upper bounds for 95% CIs. The sizes of the squares are in direct proportion to the reciprocals of the estimated standard errors of the effect sizes. HRs and 95% CIs were calculated using stratified Cox regression models, stratified by age (in 5-year intervals), sex and living areas, adjusting for education, household income, occupation, marital status, alcohol consumption, smoking status, passive smoking status, physical activity levels, primary cooking fuel, primary heating fuel usage, and consumption frequency of fresh fruits, fresh vegetables, meat, general obesity and abdominal obesity, as well as self-rated health status, prevalent hypertension and prevalent diabetes at baseline. *P < 0.05; #False discovery rate < 0.05. Abbreviations: COPD, chronic obstructive pulmonary disease; HR, hazard ratio; CI, confidence interval

COPD patients at baseline were observed to be associated with a higher risk of subsequent digestive diseases after correction for multiple testing, with a hazard ratio (HR) and 95% confidence interval (CI) of 1.05 (1.03–1.08) (Fig. 2 and Supplementary Table 8). For specific disease types, elevated risks of gastrointestinal inflammation (1.11[1.07–1.15]), abdominal pain (1.20[1.12–1.28]), and disorders of gallbladder (1.06[1.01–1.11]) were found among COPD-patients compared to non-COPD participants. Among them, two associations reached the study-wide significance level (Fig. 2). Besides, digestive diseases including gastrointestinal inflammation (incidence rate ratio[95% CI]: 1.07[1.02–1.13]), and cholecystitis (1.10[1.01–1.20]) were observed to occur more frequently among COPD patients (Supplementary Table 8) with a nominal significance.

In subgroup analyses, an interaction between sex and COPD on overall digestive diseases was identified, with the effects being marginally greater among females than males. No modification effects of other factors were observed (Supplementary Figs. 2–6).

### Associations of airflow obstruction severity in COPD at baseline with selected digestive diseases

**Fig. 3:**
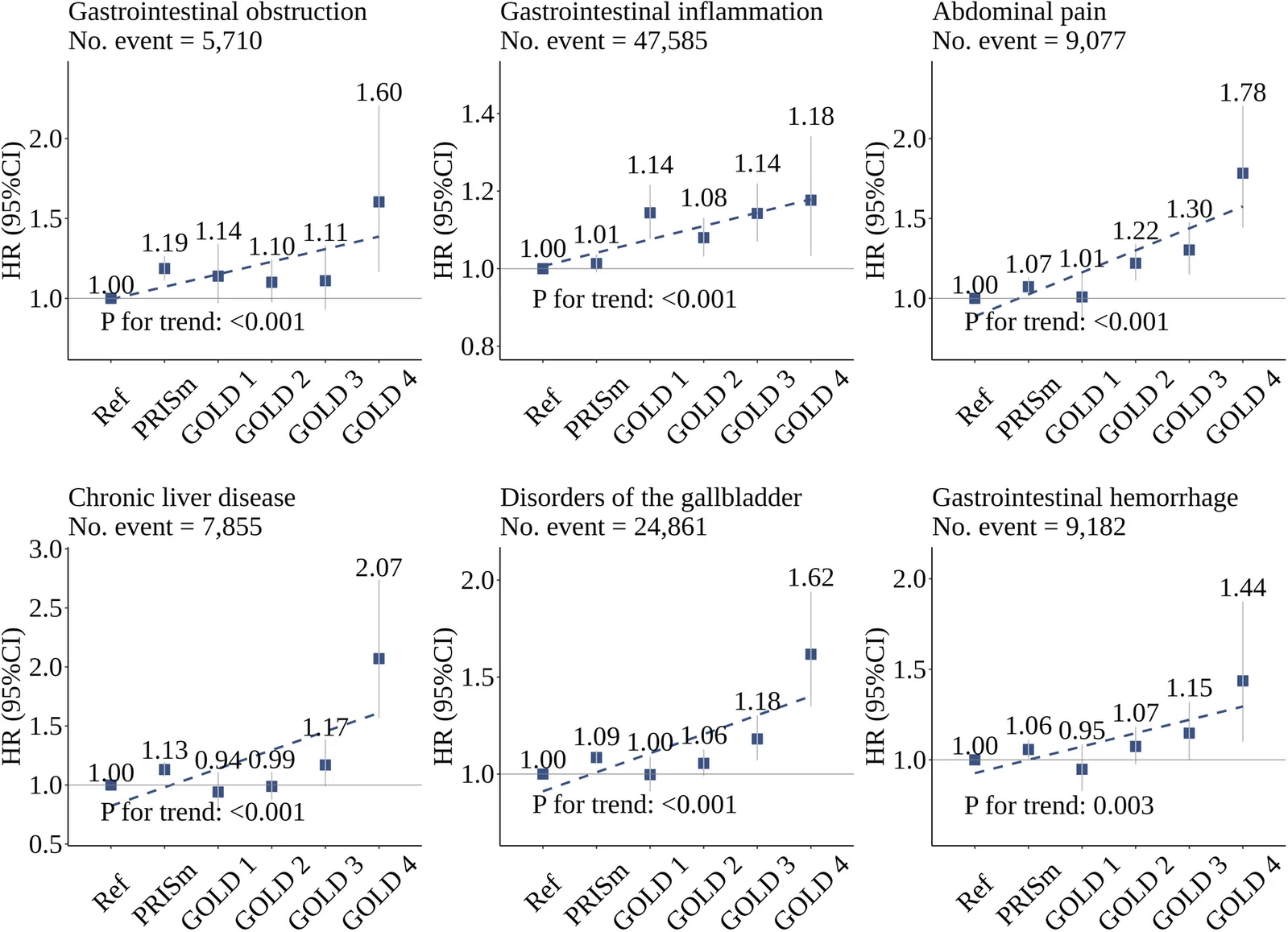
Associations of airflow obstruction severity at baseline with selected digestive diseases. The centers of the squares represent the point estimates for HRs, and the edges represent the lower and upper bounds for 95% CIs. The dashed lines represent the slope from a linear regression. Airflow obstruction severity were classified according to the GOLD 2007 criteria(GOLD stage 1, mild: FEV_1_ ≥ 80% predicted; GOLD stage 2, moderate: 50% ≤ FEV_1_ < 80% predicted; GOLD stage 3, severe: 30% ≤ FEV_1_ < 50% predicted; and GOLD stage 4, very severe: FEV_1_ < 30% predicted). PRISm represented FEV_1_ < 80% predicted among Non-COPD individuals. Using non-COPD participants with FEV_1_ ≥ 80% predicted as the reference group, HRs and 95% CIs were calculated using stratified Cox regression models, stratified by age (in 5-year intervals), sex and living areas, adjusting for education, household income, occupation, marital status, alcohol consumption, smoking status, passive smoking status, physical activity levels, primary cooking fuel, primary heating fuel usage, and consumption frequency of fresh fruits, fresh vegetables, meat, general obesity and abdominal obesity, as well as self-rated health status, prevalent hypertension and prevalent diabetes at baseline. *P* values for trend were calculated by treating the multiple categorical variables representing the airflow obstruction severity as a continuous variable in the above regression models. Abbreviations: COPD, chronic obstructive pulmonary disease; HR, hazard ratio; CI, confidence interval; FEV_1_, forced expiratory volume in one second; PRISm, preserved ratio impaired spirometry

Compared to non-COPD participants with FEV_1_ ≥ 80% predicted, a dose–response association between airflow obstruction severity and the incidence of the overall digestive diseases (Supplementary Tables 9–10, *P* for trend: < 0.001) was observed, and COPD patients at GOLD stage 4 were associated with the highest risk (1.30 [1.19–1.42]). Similar trend associations were found for gastrointestinal obstruction, gastrointestinal inflammation, abdominal pain, chronic liver disease, disorders of the galbladder(*P* values for trend: < 0.001), and gastrointestinal hemorrhage(*P* for trend: 0.003), as well as some of their subtypes (Fig. 3 and Supplementary Tables 9–10). The HRs for GOLD stage 4 with these diseases ranged from 1.18 (gastrointestinal inflammation; 95%CI: 1.03–1.34) to 2.07 (chronic liver disease; 1.57–2.74). Associations between GOLD stage 4 and fibrosis and cirrhosis of liver (1.98[1.33–2.96]), fatty liver disease (2.05[1.25–3.37]), and hepatic failure (3.59[1.46–8.83]) were also identified (Supplementary Tables 9–10), although no trend association was observed. Notably, non-COPD participants at the PRISm stage were found to be associated with higher risks of the overall digestive diseases (1.05[1.03–1.06]), and other five digestive disease types (Supplementary Tables 9–10). In subgroup analyses by smoking status, no meaningful interactions were identified (Supplementary Table 11).

Results for sensitivity analyses are shown in Supplementary Figs. 7 to 12, and Supplementary Table 13. The COPD-associated disease types and the effect sizes were almost the same as those in the primary analyses.

### Associations of genetic-predicted COPD with selected digestive diseases

**Fig. 4:**
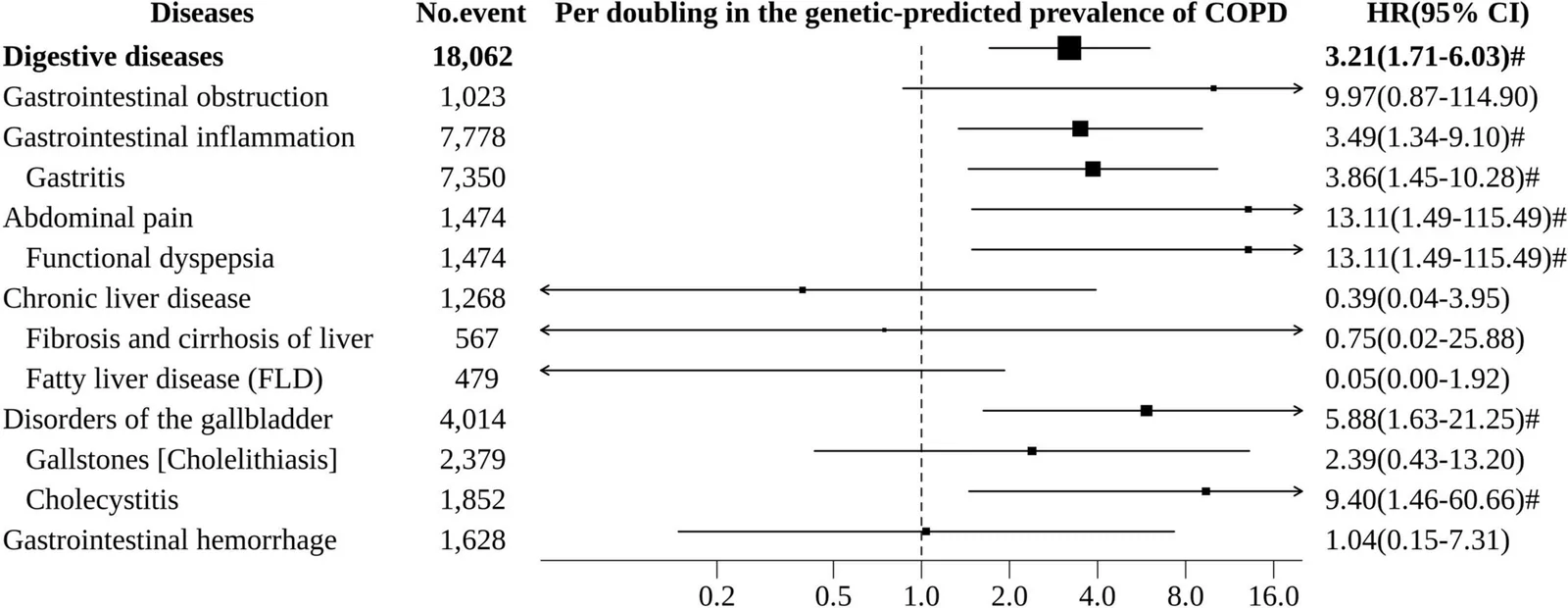
Associations of genetically predicted COPD with selected digestive diseases. The centers of the squares represent the point estimates for HRs, and the edges represent the lower and upper bounds for 95% CIs. The sizes of the squares are in direct proportion to the reciprocals of the estimated standard errors of the effect sizes. Genetically predicted probabilities of COPD were calculated using genetic IVs(GRSs) in logistic regression models adjusting for the top ten principal components and genetic array. The GRS used was ‘CKB GRS 5e8’ calculated from lead SNPs selected from the CKB-GWAS.. HRs and 95% CIs were computed using stratified Cox regression models, stratified by age (in 5-year intervals), sex and living areas, adjusting for education, household income, occupation, marital status, alcohol consumption, smoking status, passive smoking status, physical activity levels, primary cooking fuel, primary heating fuel usage, and consumption frequency of fresh fruits, fresh vegetables, meat, general obesity and abdominal obesity, self-rated health status, prevalent hypertension and prevalent diabetes at baseline, as well as the top ten principal components and genetic arrays. *P < 0.05; #*False discovery rate* < 0.05. Abbreviations: COPD, chronic obstructive pulmonary disease; HR, hazard ratio; CI, confidence interval; IV, instrumental variable; GRS, genetic risk score; CKB, China Kadoorie Biobank

In the one-sample MR analysis, CKB GRS-predicted COPD were associated with higher risks of the overall ‘GI’-coded digestive diseases (The effect size for the average change in the outcome per doubling in the prevalence of the exposure: 3.21 [1.71–6.03]), gastrointestinal inflammation (3.49 [1.34–9.10]), abdominal pain (13.11 [1.49–115.49]), and disorders of the gallbladder (5.88 [1.63–21.25]) at the study-wide significance level (Fig. 4 and Supplementary Table 14). Among them, three associations were validated using IVs constructed from the meta-GWAS at the study-wide significance level (Supplementary Fig. 13). The sensitivity analyses without adjusting for BMI, abdominal obesity, prevalent hypertension, and prevalent diabetes using revealed similar results (Supplementary Fig. 14). The 4 associations were also validated using IVs only derived from BBJ statistics (Supplementary Fig. 15–16).

## Discussion

Based on a large sample of the general population from China, our study presents comprehensive results on the associations between COPD and an increased risk of subsequent overall digestive diseases and several disease subtypes, and the MR analysis supports a potential causal role of COPD on them.

In general, our study extended the spectrum of COPD-related digestive diseases in the Chinese population. We noted that abdominal pain, primarily attributed to functional dyspepsia, was the disease most strongly associated with COPD in our main analysis. It is important to highlight that abdominal pain requiring inpatient care likely reflects a more severe clinical presentation and may significantly impair patients' quality of life [21]. Studies involving COPD patients from Taiwan identified higher risks for future functional dyspepsia and peptic ulcer among COPD patients [22, 23], which aligned with our results. Associations of gastritis and cholecystitis with COPD have been observed in case–control studies but rarely discussed in prospective studies previously [6], and our study filled the evidence gap. For MR analyses, one MR study found that COPD is weakly associated with peptic ulcer disease and is not causally related to functional dyspepsia [11]. However, the IV selection procedures were based on European ancestry, which may restrict their generalizability to other ancestries. Through using Chinese-specific IVs, our MR analyses supported most of the associations found in observational studies. It must be acknowledged that using IVs from the same population may overestimate the effects of exposures in our MR analysis. But the agreement between the CKB-derived and BBJ-derived instruments, derived from completely independent GWAS, argues against substantial bias in our primary one-sample MR analysis and may partially alleviates this concern. However, we did not observe higher risks of gastroesophageal reflux disease and inflammatory bowel disease among COPD patients. Population-based studies have reported that gastroesophageal reflux disease and inflammatory bowel disease are common COPD comorbidities [8, 24, 25], but this was not seen in our study. We assume that the null findings may partly reflect the inpatient nature of our data, which could have missed milder outpatient-managed cases. In addition, the lack of bronchodilator testing may lead to misclassification of asthma or asthma-COPD overlap as COPD. Although our results remained robust after a sensitivity analysis excluding self-reported asthma, the possibility of asthma-COPD overlap may still remain. Therefore, future studies using post-bronchodilator confirmation are encouraged to further validate these associations.

Tobacco smoking has been considered a vital confounder for COPD and digestive diseases, but our results showed that the associations might be partly independent of it [1, 26]. In our prospective analyses, the associations still remained after carefully adjusting for smoking status and passive smoking. Besides, modification effects of smoking status were not identified among both the entire study population and participants without self-reported asthma, indicating that patients of non-smoking-related COPD types would also have higher risks of subsequent digestive diseases. Moreover, results from the MR analyses support the potential direct causal role of COPD on digestive diseases. Therefore, proper interventions should also be taken to prevent digestive diseases in COPD patients without prior exposure to tobacco smoking.

The GOLD criteria are applied to track the severity of COPD in practice [27]. Our study demonstrated that the GOLD criteria might also be effective in the management of COPD-related digestive diseases, especially for several chronic liver diseases. The grading of the airflow obstruction severity led to the identification of an increased number of COPD-related digestive diseases in our study, including chronic hepatitis, fibrosis, cirrhosis of the liver, fatty liver disease, and hepatic failure. Previous evidence on the interactions between liver diseases and COPD is deemed to be weak [9], though, one study conducted in Shanghai, China, observed that impaired lung function is associated with non-alcoholic fatty liver diseases independent of metabolic risk factors [28], which supports our findings.

Several mechanisms have been proposed to explain the associations between COPD and digestive diseases. First and most importantly, the dose–response links between COPD severity and gastrointestinal inflammation reinforced the “spillover of inflammation” hypothesis. COPD has long been recognized as an inflammatory lung disease [29], resulting from the risk factor-induced inflammatory reactions and subsequently generating inflammation even after the removal of risk factors. Elevated levels of systemic inflammation factors have been documented in COPD patients [30], and the degree of systemic inflammation is closely related to impaired lung function in COPD patients. The spillover of inflammatory mediators into the circulating system can trigger cross-organ inflammation responses, resulting in the disruption of the integrity of the intestinal mucosal barrier and inflammatory cell infiltration [31], which may consequently lead to gastritis, peptic ulcer, cholecystitis, and functional dyspepsia. Inflammation-induced disease-related malnutrition has also been identified to prevent patients from ingesting sufficient nutrition and thus promoting further inflammatory reactions and functional changes [32]. Secondly, the intestinal microbiome has been indicated to play a role in the ‘lung-gut’ axis [33]. Compositional changes of specific microbiota were associated with the severity of COPD and were accompanied by epithelial defense and proinflammatory response changes [34]. Thirdly, some medications, primarily used in more severe stages of the disease, are known to have specific gastrointestinal side effects, such as the potential to induce peptic ulcers, bleeding, and other digestive tract disorders [35]. Although we adjusted for aspirin use in the sensitivity analysis, information on COPD treatment, particularly inhaled or systemic corticosteroids, was not available in our database and thus could not be accounted for*.*

In addition, our results invite further discussion of the mechanisms underlying the associations between COPD severity and chronic liver diseases also need further discussions. The inflammatory visceral fat depots have been recognized as one of the pathological changes among COPD patients [30], and the accumulated visceral fat could result in free fatty acids depositing in the liver [31], potentially leading to fatty liver disease. Besides, chronic hypoxemia is commonly seen in COPD patients, particularly in more severe stages [36]. Under long-term hypoxic conditions, a metabolic shift from fat oxidation to fat synthesis and intensified hepatic oxidative stress could promote the development of FLD and even fibrosis [37]. Intestinal dysbiosis accompanied by the progression of COPD may induce the translocation of bacteria and their products, which could activate additional innate immune responses [38].

Notably, our study also bridged the associations between PRISm and several digestive diseases, including gastrointestinal obstruction, abdominal pain, several chronic liver diseases, and disorders of the gallbladder, while such links were not observed for GOLD 1 stage COPD. Non-COPD individuals at the PRISm stage are defined as having normal FEV_1_/FVC with non-obstructive pulmonary function abnormalities characterized by a decrease in FEV_1_. Prior epidemiological evidence have shown that individuals at the PRISm stage exhibit a higher burden of comorbidities compared to those with normal lung function, including: diabetes, hypertension, ischemic stroke, ischemic heart disease [39, 40]. This may be attributable to higher levels of inflammatory mediators including C reactive protein induced by impaired airway patency [41]. Our findings further extend this pattern to digestive morbidity, underscoring the clinical relevance of lung function impairment even below the diagnostic threshold for COPD. Thus, monitoring individuals with milder or non-obstructive lung function impairment, including those with PRISm, may offer an opportunity for early preventive strategies against specific digestive diseases. However, PRISm is a heterogeneous condition that may encompass early-stage COPD, restrictive lung disease, obesity-related lung restriction, or other cardiometabolic disturbances. Given that post-bronchodilator spirometry and more specific chest testing results were absent at baseline, it is difficult to determine the true etiology of PRISm. Therefore, whether PRISm could represent the early stage of COPD in our results should also be interpreted with caution.

The strengths of our study include the large sample size from the Chinese population, the long-term and high-quality follow-up of digestive diseases, the utilization of Chinese-specific genetic IVs in MR analyses, and the combination of observational studies and MR analyses. Meanwhile, several limitations have to be acknowledged. First and foremost, post-bronchodilator spirometry was not done at baseline, which could result in the overdiagnosis of COPD. Although pre-bronchodilator FEV_1_/FVC was a strong predictor for future COPD development [42], the risks of misclassification of COPD still remain, and the results should be interpreted with more caution. Second, as discussed above, the lack of data limited our understanding of how such pharmacological treatments may independently influence digestive outcomes, such as ulceration and bleeding. However, we tried to conduct the main analysis among participants who have reported no medication history of aspirin usage and the results remained consistent. Third, the GOLD criteria (GOLD 2007) we used only utilized the spirometry results to specify the severity of airflow obstruction, which ignored the exacerbation history and symptom burden used in GOLD 2011 and GOLD 2017. However, studies comparing the prediction ability of the three GOLD criteria discovered that GOLD 2007 was a better predictor for predicting COPD hospitalization and all-cause mortality [43]. Fourth, general obesity and abdominal obesity, prevalent hypertension, and prevalent diabetes might be on the causal pathway from COPD to digestive diseases, and the adjustment for these factors may lead to the underestimation of the total effect of COPD. However, sensitivity analyses excluding these factors in both prospective and MR analyses yielded nearly identical results, indicating minimal impact of these factors on our primary models. Fifth, digestive diseases were ascertained through hospitalization or death records only. Mild cases that only visit the outpatient clinic may not be captured. Therefore, our findings are generalizable to moderate-to-severe digestive diseases requiring inpatient care rather than all community-diagnosed cases.

Several limitations should also be acknowledged for the MR analyses. First, the genetic instruments for COPD were derived from the same CKB cohort used for the outcome analysis, which may introduce bias due to the inflation of genetic effect estimates when variants are selected based on significance thresholds within the same sample. However, sensitivity analyses using IVs from only the BBJ GWAS and the CKB and BBJ meta-GWAS revealed nearly identical results, reflecting weak bias in our results. Second, the assumption of no horizontal pleiotropy is challenging to verify in a one-sample setting. Although we have tested the associations of GRSs with covariates, we cannot rule out the possibility that genetic variants influence digestive diseases through other pathways independent of COPD. Third, compared with two-sample MR, one-sample MR generally has lower statistical power, and the confidence intervals were wider in our analyses. However, there were not sufficient summary statistics for all kinds of digestive diseases available to conduct two-sample MR. Finally, while MR provides evidence consistent with a causal relationship and reduces confounding and reverse causation, the confidence intervals in the MR analysis were wide for some diseases, and it does not prove causality definitively. Besides, the strengths of the two GRSs constructed from the BBJ GWAS were relatively moderate, which warrants cautious interpretation. Therefore, the results should be interpreted as genetic evidence supporting a potential causal role of COPD in digestive diseases, and as supplementary evidence to the primary observational findings, rather than conclusive proof.

In conclusion, our study demonstrates that prevalent COPD is associated with a higher risk of diverse digestive diseases, and applying severity classification criteria is also helpful for managing COPD-related digestive diseases. With the development of multi-omics, including genomics, metagenomics, and proteomics, the mechanisms linking COPD and the digestive system could be further understood.

## Supplementary Information

Additional file 1.

## Data Availability

The China Kadoorie Biobank (CKB) is a global resource for the investigation of lifestyle, environmental, blood biochemical and genetic factors as determinants of common diseases. The CKB study group is committed to making the cohort data available to the scientific community in China, the UK and worldwide to advance knowledge about the causes, prevention and treatment of disease. For detailed information on what data is currently available to open access users and how to apply for it, visit: https://www.ckbiobank.org/data-access Researchers who are interested in obtaining the raw data from the China Kadoorie Biobank study that underlines this paper should contact pdc@kscdc.net. A research proposal will be requested to ensure that any analysis is performed by bona fide researchers and—where data is not currently available to open access researchers—is restricted to the topic covered in this paper.

## References

[1] Venkatesan P. GOLD COPD report: 2024 update. Lancet Respir Med. 2024;12:15–6. 10.1016/S2213-2600(23)00461-7 38061380

[2] Global burden of 369 diseases and injuries in 204 countries and territories, 1990–2019: a systematic analysis for the Global Burden of Disease Study 2019. Lancet. 2020;396:1204–1222. 10.1016/S0140-6736(20)30925-9 PMC7567026 33069326

[3] Adeloye D, Song P, Zhu Y, Campbell H, Sheikh A, Rudan I. Global, regional, and national prevalence of, and risk factors for, chronic obstructive pulmonary disease (COPD) in 2019: a systematic review and modelling analysis. Lancet Respir Med. 2022;10:447–58. 10.1016/S2213-2600(21)00511-7 PMC9050565 35279265

[4] Chen S, Kuhn M, Prettner K, Yu F, Yang T, Bärnighausen T, et al. The global economic burden of chronic obstructive pulmonary disease for 204 countries and territories in 2020–50: a health-augmented macroeconomic modelling study. Lancet Glob Health. 2023;11:e1183–93. 10.1016/S2214-109X(23)00217-6 PMC10369014 37474226

[5] Rabe KF, Watz H. Chronic obstructive pulmonary disease. Lancet. 2017;389:1931–40. 10.1016/S0140-6736(17)31222-9 28513453

[6] Wang L, Cai Y, Garssen J, Henricks PAJ, Folkerts G, Braber S. The bidirectional gut-lung axis in chronic obstructive pulmonary disease. Am J Respir Crit Care Med. 2023;207:1145–60. 10.1164/rccm.202206-1066TR PMC10161745 36883945

[7] Rutten EP, Spruit MA, Franssen FM, Buurman WA, Wouters EF, Lenaerts K. GI symptoms in patients with COPD. Chest. 2014;145:1437–8. 10.1378/chest.14-0285 24889455

[8] Brassard P, Vutcovici M, Ernst P, Patenaude V, Sewitch M, Suissa S, et al. Increased incidence of inflammatory bowel disease in Québec residents with airway diseases. Eur Respir J. 2015;45:962–8. 10.1183/09031936.00079414 25406447

[9] Lonardo A, Nascimbeni F, Ponz de Leon M: Nonalcoholic fatty liver disease and COPD: is it time to cross the diaphragm? Eur Respir J. 2017;49. 10.1183/13993003.00546-2017 28596428

[10] Chun EM, Kim SW, Lim SY. Prevalence of colorectal adenomatous polyps in patients with chronic obstructive pulmonary disease. Int J Chron Obstruct Pulmon Dis. 2015;10:955–60. 10.2147/COPD.S83341 PMC4441357 26028968

[11] Shen Z, Qiu B, Chen L, Zhang Y. Common gastrointestinal diseases and chronic obstructive pulmonary disease risk: a bidirectional Mendelian randomization analysis. Front Genet. 2023;14:1256833. 10.3389/fgene.2023.1256833 PMC10690629 38046045

[12] Negewo NA, Gibson PG, McDonald VM. COPD and its comorbidities: impact, measurement and mechanisms. Respirology. 2015;20:1160–71. 10.1111/resp.12642 26374280

[13] Hurst JR, Vestbo J, Anzueto A, Locantore N, Müllerova H, Tal-Singer R, et al. Susceptibility to exacerbation in chronic obstructive pulmonary disease. N Engl J Med. 2010;363:1128–38. 10.1056/NEJMoa0909883 20843247

[14] Chen Z, Chen J, Collins R, Guo Y, Peto R, Wu F, et al. China Kadoorie Biobank of 0.5 million people: survey methods, baseline characteristics and long-term follow-up. Int J Epidemiol. 2011;40:1652–66. 10.1093/ije/dyr120 PMC3235021 22158673

[15] Kurmi OP, Li L, Smith M, Augustyn M, Chen J, Collins R, et al. Regional variations in the prevalence and misdiagnosis of air flow obstruction in China: baseline results from a prospective cohort of the China Kadoorie Biobank (CKB). BMJ Open Respir Res. 2014;1:e000025. 10.1136/bmjresp-2014-000025 PMC4212802 25478177

[16] Quanjer PH, Stanojevic S, Cole TJ, Baur X, Hall GL, Culver BH, et al. Multi-ethnic reference values for spirometry for the 3–95-yr age range: the global lung function 2012 equations. Eur Respir J. 2012;40:1324–43. 10.1183/09031936.00080312 PMC3786581 22743675

[17] Huang J, Li W, Sun Y, Huang Z, Cong R, Yu C, et al. Preserved ratio impaired spirometry (PRISm): a global epidemiological overview, radiographic characteristics, comorbid associations, and differentiation from chronic obstructive pulmonary disease. Int J Chron Obstruct Pulmon Dis. 2024;19:753–64. 10.2147/COPD.S453086 PMC10949882 38505581

[18] Shuey MM, Stead WW, Aka I, Barnado AL, Bastarache JA, Brokamp E, Campbell M, Carroll RJ, Goldstein JA, Lewis A, et al: Next-generation phenotyping: introducing phecodeX for enhanced discovery research in medical phenomics. Bioinformatics. 2023;39. 10.1093/bioinformatics/btad655 PMC10627409 37930895

[19] Willer CJ, Li Y, Abecasis GR. METAL: fast and efficient meta-analysis of genomewide association scans. Bioinformatics. 2010;26:2190–1. 10.1093/bioinformatics/btq340 PMC2922887 20616382

[20] Sakaue S, Kanai M, Tanigawa Y, Karjalainen J, Kurki M, Koshiba S, et al. A cross-population atlas of genetic associations for 220 human phenotypes. Nat Genet. 2021;53:1415–24. 10.1038/s41588-021-00931-x PMC12208603 34594039

[21] Broeders B, Carbone F, Balsiger LM, Schol J, Raymenants K, Huang I, et al. Review article: functional dyspepsia-a gastric disorder, a duodenal disorder or a combination of both? Aliment Pharmacol Ther. 2023;57:851–60. 10.1111/apt.17414 36859629

[22] Chiu YC, Chang WP, Tang GJ, Lan TY, Lee KY, Su VY. Chronic obstructive pulmonary disease is associated with a higher risk of functional gastrointestinal disorders. Respir Med. 2022;197:106833. 10.1016/j.rmed.2022.106833 35427844

[23] Huang KW, Kuan YC, Chi NF, Huang YH, Luo JC, Chien LN. Chronic obstructive pulmonary disease is associated with increased recurrent peptic ulcer bleeding risk. Eur J Intern Med. 2017;37:75–82. 10.1016/j.ejim.2016.09.020 27727075

[24] Kim J, Lee JH, Kim Y, Kim K, Oh YM, Yoo KH, et al. Association between chronic obstructive pulmonary disease and gastroesophageal reflux disease: a national cross-sectional cohort study. BMC Pulm Med. 2013;13:51. 10.1186/1471-2466-13-51 PMC3750392 23927016

[25] Broers C, Tack J, Pauwels A. Review article: gastro-oesophageal reflux disease in asthma and chronic obstructive pulmonary disease. Aliment Pharmacol Ther. 2018;47:176–91. 10.1111/apt.14416 29193245

[26] Larsson SC, Burgess S. Appraising the causal role of smoking in multiple diseases: a systematic review and meta-analysis of Mendelian randomization studies. EBioMedicine. 2022;82:104154. 10.1016/j.ebiom.2022.104154 PMC9278068 35816897

[27] Rabe KF, Hurd S, Anzueto A, Barnes PJ, Buist SA, Calverley P, et al. Global strategy for the diagnosis, management, and prevention of chronic obstructive pulmonary disease: GOLD executive summary. Am J Respir Crit Care Med. 2007;176:532–55. 10.1164/rccm.200703-456SO 17507545

[28] Qin L, Zhang W, Yang Z, Niu Y, Li X, Lu S, et al. Impaired lung function is associated with non-alcoholic fatty liver disease independently of metabolic syndrome features in middle-aged and elderly Chinese. BMC Endocr Disord. 2017;17:18. 10.1186/s12902-017-0168-4 PMC5361719 28330472

[29] Christenson SA, Smith BM, Bafadhel M, Putcha N. Chronic obstructive pulmonary disease. Lancet. 2022;399:2227–42. 10.1016/S0140-6736(22)00470-6 35533707

[30] Hlapčić I, Belamarić D, Bosnar M, Kifer D, Vukić Dugac A, Rumora L: Combination of Systemic Inflammatory Biomarkers in Assessment of Chronic Obstructive Pulmonary Disease: Diagnostic Performance and Identification of Networks and Clusters. Diagnostics (Basel). 2020;10. 10.3390/diagnostics10121029 PMC7760570 33266187

[31] Keely S, Hansbro PM. Lung-gut cross talk: a potential mechanism for intestinal dysfunction in patients with COPD. Chest. 2014;145:199–200. 10.1378/chest.13-2077 24493496

[32] Wunderle C, Stumpf F, Schuetz P. Inflammation and response to nutrition interventions. JPEN J Parenter Enter Nutr. 2024;48:27–36. 10.1002/jpen.2534 38193635

[33] Song Z, Meng Y, Fricker M, Li X, Tian H, Tan Y, et al. The role of gut-lung axis in COPD: pathogenesis, immune response, and prospective treatment. Heliyon. 2024;10:e30612. 10.1016/j.heliyon.2024.e30612 PMC11089359 38742057

[34] Ramsheh MY, Haldar K, Esteve-Codina A, Purser LF, Richardson M, Müller-Quernheim J, et al. Lung microbiome composition and bronchial epithelial gene expression in patients with COPD versus healthy individuals: a bacterial 16S rRNA gene sequencing and host transcriptomic analysis. Lancet Microbe. 2021;2:e300–10. 10.1016/S2666-5247(21)00035-5 35544166

[35] Oray M, Abu Samra K, Ebrahimiadib N, Meese H, Foster CS. Long-term side effects of glucocorticoids. Expert Opin Drug Saf. 2016;15:457–65. 10.1517/14740338.2016.1140743 26789102

[36] Kent BD, Mitchell PD, McNicholas WT. Hypoxemia in patients with COPD: cause, effects, and disease progression. Int J Chron Obstruct Pulmon Dis. 2011;6:199–208. 10.2147/COPD.S10611 PMC3107696 21660297

[37] Fuster-Martínez I, Bernal-Monterde V, Bidault G, Arbonés-Mainar JM, Vidal-Puig A: Hypoxia in MASLD: a spatial determinant of the pathogenesis. Trends Mol Med. 2026. 10.1016/j.molmed.2025.12.008. PMC7618922 41545235

[38] Patil S, Doshi G: Gut Microbiota in the Hepato-Cardiorenal Axis: Microbial Metabolites, Inflammation, and Emerging Therapeutic Targets. Curr Pharm Des 2026. 10.2174/0113816128413464251209115653 41572742

[39] Wan ES, Castaldi PJ, Cho MH, Hokanson JE, Regan EA, Make BJ, et al. Epidemiology, genetics, and subtyping of preserved ratio impaired spirometry (PRISm) in COPDGene. Respir Res. 2014;15:89. 10.1186/s12931-014-0089-y PMC4256936 25096860

[40] Kim J, Lee CH, Lee HY, Kim H. Association between comorbidities and preserved ratio impaired spirometry: using the Korean national health and nutrition examination survey IV-VI. Respiration. 2022;101:25–33. 10.1159/000517599 PMC8820418 34320510

[41] Yang X, Liao J, Zhu S, Zhang C, Ma X, Zhang C, Wang Y, Sun K, Wang G: Association of high-sensitivity CRP and FEV1%pred: a study on non-pulmonary disease in a population in Beijing, China. BMJ Open Respir Res. 2024;11. 10.1136/bmjresp-2023-001699 PMC10941139 38479820

[42] Tan DJ, Lodge CJ, Walters EH, Bui DS, Pham J, Lowe AJ, et al. Can we use lung function thresholds and respiratory symptoms to identify pre-chronic obstructive pulmonary disease? A prospective, population-based cohort study. Am J Respir Crit Care Med. 2024;209:1431–40. 10.1164/rccm.202212-2330OC 38236192

[43] Brusse-Keizer M, Klatte M, Zuur-Telgen M, Koehorst-Ter Huurne K, van der Palen J, VanderValk P. Comparing the 2007 and 2011 GOLD classifications as predictors of all-cause mortality and morbidity in COPD. COPD. 2007;14:7–14. 10.1080/15412555.2016.1206875 27442500

