# Supplementary material for: Chronic obstructive pulmonary disease and risk of digestive diseases: observational and mendelian randomization analyses in the China Kadoorie Biobank
Source: Respir Res. 2026 Jul 17;27:395. doi: 10.1186/s12931-026-03825-w (PMC13660256; doi:10.1186/s12931-026-03825-w)
Supplement: Supplementary file 1 [file 12931_2026_3825_MOESM1_ESM.docx]

# Associations between COPD and digestive diseases: evidence from the China Kadoorie Biobank

# Supplementary materials

**Supplementary Table 1. Overview of the population characteristics of the CKB population**

**Supplementary Table 2. The detailed information of the plus inclusion criteria, mapping rules from ICD-10 code to phecodeX, participants included, number of events occurred, and incident densities for the selected digestive diseases**

**Supplementary Table 3. Detailed grouping information of the included covariates**

**Supplementary Text 1. The methods for the genome-wide associations studies on COPD in the CKB population, IV selection procedure in the CKB and BBJ population**

**Supplementary Figure 1. The Manhattan plot and Q-Q plots for GWAS results in the CKB population and meta-GWAS results in the CKB and BBJ populations**

**Supplementary Table 4. The summary information for the SNPs used to construct CKB and meta genetic risk scores**

**Supplementary Table 5. The summary information for the SNPs used to construct BBJ genetic risk scores**

**Supplementary Table 6. The associations between SNPs used to construct genetic risk scores and covariates**

**Supplementary Table 7. The associations between constructed genetic risk scores and covariates**

**Supplementary Table 8.** **Associations of prevalent COPD at baseline with selected digestive diseases**

**Supplementary Table 9. Associations of prevalent COPD at baseline with the frequency of occurrence for the selected digestive diseases**

**Supplementary Figure 2. Associations of prevalent COPD at baseline with selected digestive diseases in old (**≥**60 years old) and young (**<**60 years old) participants**

**Supplementary Figure 3. Associations of prevalent COPD at baseline with selected digestive diseases in male and female participants**

**Supplementary Figure 4. Associations of prevalent COPD at baseline with selected digestive diseases in low BMI (BMIl, <24kg/m^2^) and high BMI (BMIh, ≥24kg/m^2^) participants**

**Supplementary Figure 5. Associations of prevalent COPD at baseline with selected digestive diseases in non-smokers and ever-smokers**

**Supplementary Figure 6. Associations of prevalent COPD at baseline with selected digestive diseases in non-daily drinkers and daily drinkers**

**Supplementary Table 10. The detailed information on the incident densities of the selected digestive diseases**

**Supplementary Table 11. Associations of airflow obstruction severity in COPD at baseline with selected digestive diseases**

**Supplementary Table 12. Associations of airflow obstruction severity in COPD at baseline with selected digestive diseases in never-smokers and ever-smokers**

**Supplementary Figure 7. Associations of prevalent COPD at baseline with selected digestive diseases after excluding participants whose follow-up time was less than 2 years**

**Supplementary Figure 8. Associations of prevalent COPD at baseline with selected digestive diseases after additionally adjusting for anxiety**

**Supplementary Figure 9. Associations of prevalent COPD at baseline with selected digestive diseases after excluding self-reported asthma at baseline**

**Supplementary Figure 10. Associations of prevalent COPD at baseline with selected digestive diseases after excluding participants who have reported the history of aspirin usage at baseline**

**Supplementary Figure 11. Associations of prevalent COPD at baseline with selected digestive diseases without adjusting for BMI, abdominal obesity, prevalent hypertension, and prevalent diabetes**

**Supplementary Figure 12. Associations of prevalent COPD at baseline with selected digestive diseases in never-smokers without self-reported asthma and ever-smokers without self-reported asthma**

**Supplementary Table 13. Sensitivity analysis accounting for competing risk of death for significant digestive outcomes**

**Supplementary Table 14. Associations of genetically predicted COPD with selected digestive diseases**

**Supplementary Figure 13. Associations of genetically predicted COPD with selected digestive diseases using IVs from the meta-GWAS**

**Supplementary Figure 14. Associations of genetically predicted COPD with selected digestive diseases using IVs from the CKB-GWAS without adjusting for BMI, abdominal obesity, prevalent hypertension, and prevalent diabetes**

**Supplementary Figure 15. Associations of genetically predicted COPD with selected digestive diseases using IVs from the BBJ-GWAS**

**Supplementary Figure 16. Associations of genetically predicted COPD with selected digestive diseases using IVs from the BBJ-GWAS after excluding two heterogeneous SNPs**

|  | **Overall** | **Male** | **Female** |
| --- | --- | --- | --- |
| N | 512724 | 210205 (41.0) | 302519 (59.0) |
| Age (mean [SD]) | 52.03 (10.68) | 52.85 (10.91) | 51.46 (10.48) |
| Residing areas (%) |  |  |  |
| Qingdao (urban) | 35508 (6.9) | 15624 (7.4) | 19884 (6.6) |
| Harbin (urban) | 57555 (11.2) | 23253 (11.1) | 34302 (11.3) |
| Haikou (urban) | 29685 (5.8) | 10794 (5.1) | 18891 (6.2) |
| Suzhou (urban) | 53269 (10.4) | 22366 (10.6) | 30903 (10.2) |
| Liuzhou (urban) | 50174 (9.8) | 19321 (9.2) | 30853 (10.2) |
| Sichuan (rural) | 55686 (10.9) | 21315 (10.1) | 34371 (11.4) |
| Gansu (rural) | 49887 (9.7) | 19298 (9.2) | 30589 (10.1) |
| Henan (rural) | 63356 (12.4) | 27841 (13.2) | 35515 (11.7) |
| Zhejiang (rural) | 57704 (11.3) | 24027 (11.4) | 33677 (11.1) |
| Hunan (rural) | 59900 (11.7) | 26366 (12.5) | 33534 (11.1) |
| Education (%) |  |  |  |
| Primary school or below | 260365 (50.8) | 88776 (42.2) | 171589 (56.7) |
| Middle or high school | 222383 (43.4) | 104892 (49.9) | 117491 (38.8) |
| Technical school/college or above | 29976 (5.8) | 16537 (7.9) | 13439 (4.4) |
| Occupation (%) |  |  |  |
| Retired or unemployed | 160276 (31.3) | 46210 (22.0) | 114066 (37.7) |
| Agriculture or factory-related | 286319 (55.8) | 131486 (62.6) | 154833 (51.2) |
| Others | 66129 (12.9) | 32509 (15.5) | 33620 (11.1) |
| Income (%) |  |  |  |
| <10,000 CNY/year | 144734 (28.2) | 54724 (26.0) | 90010 (29.8) |
| 10,000-19,999 CNY/year | 148958 (29.1) | 59544 (28.3) | 89414 (29.6) |
| ≥20,000 CNY/year | 219032 (42.7) | 95937 (45.6) | 123095 (40.7) |
| Married (%) | 464473 (90.6) | 195299 (92.9) | 269174 (89.0) |

**Supplementary Table 1. Overview of the population characteristics of the CKB population**

**Supplementary Table 2. The detailed information of the plus inclusion criteria, mapping rules from ICD-10 code to phecodeX, participants included, number of events occurred, and incident densities for the selected digestive diseases**

| Phecodes and Diseases | Corresponding ICD-10 codes | Plus inclusion criteria^a^ | Total participants included in analyses | No. of events | Total incident density^b^ | Incident density in non-COPD participants^b^ | Incident density in COPD patients^b^ |
| --- | --- | --- | --- | --- | --- | --- | --- |
| GI_ Digestive diseases | All the digestive diseases included | Not diagnosed as having gastric ulcer, cirrhosis/chronic hepatitis, or gallstone/gallbladder disease at baseline | 455,831 | 112,392 | 18.67 | 18.29 | 24.35 |
| GI_510 Diseases of the esophagus | K20\|K21.0\|K22\|K22.0\|K22.3\|K22.4\|K22.5\|K22.6\|K22.7\|K22.8\|K22.9\|K23\|K23.0\|K23.1\|K23.8 | - | 509,550 | 2,750 | 0.36 | 0.35 | 0.45 |
| GI_511 Gastroesophageal reflux disease [GERD] | K21\|K21.0\|K21.9 | - | 509,550 | 2,080 | 0.27 | 0.27 | 0.31 |
| GI_513 Peptic ulcer | K22.1\|K25\|K25.0\|K25.1\|K25.2\|K25.3\|K25.4\|K25.5\|K25.6\|K25.7\|K25.9\|K26\|K26.0\|K26.1\|K26.2\|K26.3\|K26.4\|K26.5\|K26.6\|K26.7\|K26.9\|K27\|K27.0\|K27.1\|K27.2\|K27.3\|K27.4\|K27.5\|K27.6\|K27.7\|K27.9\|K28\|K28.0\|K28.1\|K28.2\|K28.3\|K28.4\|K28.5\|K28.6\|K28.7\|K28.9 | Not diagnosed as having a gastric ulcer at baseline | 489,600 | 7,156 | 0.97 | 0.93 | 1.50 |
| GI_513.2 Gastric ulcer | K25\|K25.0\|K25.1\|K25.2\|K25.3\|K25.4\|K25.5\|K25.6\|K25.7\|K25.9 | Not diagnosed as having a gastric ulcer at baseline | 489,600 | 4,391 | 0.59 | 0.56 | 1.01 |
| GI_514 Gastrointestinal obstruction | K22.2\|K31.5\|K40.0\|K40.3\|K41.0\|K41.3\|K42.0\|K43.0\|K43.3\|K43.6\|K44.0\|K45.0\|K46.0\|K56\|K56.0\|K56.3\|K56.4\|K56.5\|K56.6\|K56.7\|P75\|P76\|P76.0\|P76.1\|P76.2\|P76.8\|P76.9 | - | 509,550 | 5,710 | 0.74 | 0.71 | 1.22 |
| GI_517 Vascular disorders of the intestine | K55\|K55.0\|K55.1\|K55.2\|K55.3\|K55.8\|K55.9\|P77 | - | 509,550 | 237 | 0.03 | 0.03 | 0.03 |
| GI_522 Gastrointestinal inflammation | K29\|K29.0\|K29.1\|K29.2\|K29.3\|K29.4\|K29.5\|K29.6\|K29.7\|K29.8\|K29.9\|K50\|K50.0\|K50.1\|K50.8\|K50.9\|K51\|K51.0\|K51.2\|K51.3\|K51.4\|K51.5\|K51.8\|K51.9\|K52\|K52.1\|K52.2\|K52.3\|K52.8\|K52.9\|K62.6\|K63.3\|M07.4\|M07.5\|M09.1\|M09.2 | - | 509,550 | 47,585 | 6.50 | 6.32 | 9.00 |
| GI_522.1 Inflammatory bowel disease | K50\|K50.0\|K50.1\|K50.8\|K50.9\|K51\|K51.0\|K51.2\|K51.3\|K51.4\|K51.5\|K51.8\|K51.9\|K52.3\|M07.4\|M07.5\|M09.1\|M09.2 | - | 509,550 | 539 | 0.07 | 0.07 | 0.08 |
| GI_522.7 Ulceration of the lower GI tract | K62.6\|K63.3 | - | 509,550 | 115 | 0.01 | 0.01 | 0.02 |
| GI_522.8 Duodenitis | K29.8 | - | 509,550 | 259 | 0.03 | 0.03 | 0.03 |
| GI_522.9 Gastritis | K29\|K29.0\|K29.1\|K29.2\|K29.3\|K29.4\|K29.5\|K29.6\|K29.7\|K29.9 | - | 509,550 | 44,794 | 6.10 | 5.93 | 8.48 |
| GI_524 Functional intestinal disorder | K58\|K58.1\|K58.2\|K58.3\|K58.8\|K59\|K59.2\|K59.3\|K59.8\|K59.9 | - | 509,550 | 631 | 0.08 | 0.08 | 0.13 |
| GI_524.1 Irritable bowel syndrome | K58\|K58.1\|K58.2\|K58.3\|K58.8 | - | 509,550 | 172 | 0.02 | 0.02 | 0.03 |
| GI_526 Intestinal infection | A00\|A00.0\|A00.1\|A00.9\|A01\|A01.1\|A01.2\|A01.3\|A01.4\|A02\|A02.0\|A03\|A03.0\|A03.1\|A03.2\|A03.3\|A03.8\|A03.9\|A04.0\|A04.1\|A04.2\|A04.3\|A04.4\|A04.5\|A04.6\|A04.7\|A04.8\|A04.9\|A05\|A05.0\|A05.1\|A05.2\|A05.3\|A05.4\|A05.8\|A05.9\|A06\|A06.0\|A06.1\|A06.2\|A06.3\|A06.9\|A07\|A07.0\|A07.1\|A07.2\|A07.3\|A07.8\|A07.9\|A08\|A08.0\|A08.1\|A08.2\|A08.3\|A08.4\|A08.5\|A09\|A09.0\|A09.9\|A21.3\|A22.2\|B98.1 | - | 509,550 | 18,301 | 2.42 | 2.35 | 3.40 |
| GI_527 Abdominal pain | K30\|R10\|R10.0\|R10.1\|R10.2\|R10.3\|R10.4 | - | 509550 | 9,077 | 1.19 | 1.12 | 2.11 |
| GI_527.3 Functional dyspepsia | K30 | - | 509,550 | 9,077 | 1.19 | 1.12 | 2.11 |
| GI_535 Intestinal or peritoneal adhesions | K56.5\|K66.0\|N99.4 | - | 509,550 | 901 | 0.12 | 0.11 | 0.16 |
| GI_537 Abnormality of the peritoneum | A54.2\|K35.2\|K35.3\|K65\|K65.0\|K65.8\|K65.9\|K66\|K66.1\|K66.2\|K66.8\|K66.9\|K67\|K67.0\|K67.1\|K67.2\|K67.3\|K67.8\|N73.3\|N73.4\|N73.5\|P78.1 | - | 509,550 | 2,938 | 0.38 | 0.37 | 0.48 |
| GI_537.1 Peritonitis | A54.2\|K35.2\|K35.3\|K65\|K65.0\|K65.8\|K65.9\|K67.0\|K67.1\|K67.2\|K67.3\|N73.3\|N73.4\|N73.5\|P78.1 | - | 509,550 | 2,850 | 0.37 | 0.36 | 0.47 |
| GI_540 Hepatitis | B15\|B15.0\|B15.9\|B16\|B16.0\|B16.1\|B16.2\|B16.9\|B17\|B17.0\|B17.1\|B17.2\|B17.8\|B17.9\|B18\|B18.0\|B18.00\|B18.09\|B18.1\|B18.10\|B18.19\|B18.2\|B18.8\|B18.9\|B19\|B19.0\|B19.9\|B25.1\|B58.1\|B94.2\|K70.1\|K71.2\|K71.3\|K71.4\|K71.5\|K71.6\|K73\|K73.0\|K73.1\|K73.2\|K73.8\|K73.9\|K75.2\|K75.3\|K75.4\|O98.4 | Not diagnosed as having cirrhosis/chronic hepatitis at baseline | 503,388 | 636 | 0.08 | 0.08 | 0.07 |
| GI_540.1 Chronic hepatitis | B18\|B18.0\|B18.00\|B18.09\|B18.1\|B18.10\|B18.19\|B18.2\|B18.8\|B18.9\|K71.3\|K71.4\|K71.5\|K71.6\|K73\|K73.0\|K73.1\|K73.2\|K73.8\|K73.9\|K75.4 | Not diagnosed as having cirrhosis/chronic hepatitis at baseline | 503,388 | 545 | 0.07 | 0.07 | 0.06 |
| GI_542 Chronic liver disease | K70.1 | Not diagnosed as having cirrhosis/chronic hepatitis at baseline | 503,388 | 7,855 | 1.03 | 1.02 | 1.29 |
| GI_542.1 Fibrosis and cirrhosis of the liver | K70.0\|K70.2\|K70.3\|K70.4\|K71\|K71.0\|K71.1\|K71.7\|K71.8\|K71.9\|K72\|K72.0\|K72.1\|K72.9\|K74\|K74.0\|K74.1\|K74.2\|K74.3\|K74.4\|K74.5\|K74.6\|K75\|K75.8\|K75.9\|K76.0\|K76.2\|K76.6\|K76.7 | Not diagnosed as having cirrhosis/chronic hepatitis at baseline | 503,388 | 3,441 | 0.45 | 0.44 | 0.61 |
| GI_542.2 Fatty liver disease (FLD) | K70.2\|K70.3\|K71.7\|K74\|K74.0\|K74.1\|K74.2\|K74.6 | Not diagnosed as having cirrhosis/chronic hepatitis at baseline | 503,388 | 2,959 | 0.39 | 0.38 | 0.45 |
| GI_542.3 Hepatic failure | K70.0\|K76.0 | Not diagnosed as having cirrhosis/chronic hepatitis at baseline | 503,388 | 544 | 0.07 | 0.07 | 0.11 |
| GI_542.7 Chronic nonalcoholic liver disease | K70.4\|K72\|K72.0\|K72.1\|K72.9 | Not diagnosed as having cirrhosis/chronic hepatitis at baseline | 503,388 | 2,631 | 0.35 | 0.34 | 0.39 |
| GI_550 Disorders of the gallbladder | K74\|K74.0\|K74.2\|K74.6 | Not diagnosed as having gallstone/gallbladder disease at baseline | 478,685 | 24,861 | 3.52 | 3.45 | 4.48 |
| GI_550.1 Gallstones [Cholelithiasis] | K80\|K80.0\|K80.1\|K80.2\|K80.3\|K80.4\|K80.5\|K80.8\|K81\|K81.0\|K81.1\|K81.8\|K81.9\|K82\|K82.0\|K82.1\|K82.2\|K82.3\|K82.4\|K82.8\|K82.9 | Not diagnosed as having gallstone/gallbladder disease at baseline | 478,685 | 14,725 | 2.06 | 2.02 | 2.53 |
| GI_550.2 Cholecystitis | K80\|K80.0\|K80.1\|K80.2\|K80.3\|K80.4\|K80.5\|K80.8 | Not diagnosed as having gallstone/gallbladder disease at baseline | 478,685 | 11,387 | 1.59 | 1.55 | 2.15 |
| GI_557 Gastrointestinal hemorrhage | K81\|K81.0\|K81.1\|K81.8\|K81.9 | - | 509,550 | 9,182 | 1.20 | 1.15 | 1.90 |

^a^Participants with corresponding diseases at baseline were excluded to avoid potential reverse causation.

^b^Incident densities referred to the number of cases that occurred during follow-up per 1,000 person-years.

Abbreviations: COPD, chronic obstructive pulmonary disease.

**Supplementary Table 3. Detailed grouping information of the included covariates**

| Covariates | Groups |
| --- | --- |
| Education | 1)Primary school or below; 2)Middle or high school; 3)Technical school/college or above. |
| Household income | 1)<10,000 CNY/year; 2)10,000-19,999 CNY/year; 3)≥20,000 CNY/year |
| Occupation | 1)Agriculture or factory-related; 2)Others; 3)Retired or unemployed |
| Marital status | 1)Married; 2)Not married |
| Alcohol consumption | 1)Never or not weekly; 2)Ex-regular; 3)Weekly but not daily; 4)Current <15g/d; 5)Current 15–29g/d; 6)Current 30–59g/d; 7)Current ≥60g/d |
| Smoking status | 1)Never or occasional; 2)Ex-regular; 3)Current <15 cigarettes equivalent per day; 4)Current 15–24 cigarettes equivalent per day; 5)Current ≥25 cigarettes equivalent per day |
| Passive smoking | 1)No passive smoking; 2)Passive smoking <20 years; 3)Passive smoking ≥20h/d lasting ≥20 years; 4)Passive smoking <20h/d lasting ≥20 years; 5)Not available |
| Physical activity levels | Three groups by age- and sex-specific physical activity tertiles |
| Primary cooking fuel | 1)Gas and electricity as clean fuels; 2)Wood and coal as solid fuels; 3)Other fuels; 4)Not available |
| Primary heating fuel usage | 1)Central heating; 2)Gas and electricity as clean fuels; 3)Wood and coal as solid fuels; 4)Other fuels; 5)Not available |
| Consumption frequency of fresh fruits and vegetables | 1)Daily; 2)not daily |
| Consumption frequency of meat | 1)> 4 days/week; 2)≤4 days/week |
| General obesity | 1)BMI<18.5kg/m2; 2)18.5–23.9kg/m2; 3)24–27.9kg/m2; 4)≥28.0 kg/m2 |
| Abdominal obesity | A waist ≥90 cm for men and ≥85 cm for women |
| Disease status | 1)Yes at baseline; 2)No at baseline |

**Supplementary Text 1. The methods for the genome-wide associations studies on COPD in the CKB population, IV selection procedure in the CKB and BBJ population**

**Genotyping in CKB**

CKB has conducted three phases of genotyping. A custom-designed biobank array for Chinese ancestry on the Affymetrix Axiom platform was used in the first phase, covering 700K single-nucleotide polymorphisms (SNPs) and genotyping ~32,000 CKB participants. In the second and third phases, the original array was revised based on the genotyping data of the first 100 plates. The updated version, which covered ~803K SNPs, was used for genotyping ~72,000 participants. After removing participants who failed initial quality control, had a call rate of less than 95%, exhibited excess heterozygosity and homozygosity, were ancestry outliers, or had missing or withdrawn consent, a total of 100,706 participants remained.

The genotypes for each chromosome were phased using SHAPEIT. Imputation was conducted for each 5-Mb interval using IMPUTE4, based on haplotypes derived from the 1000 Genomes Phase 3.

**GWAS and IVs selection on COPD in the CKB population**

Based on the population included in the observational studies, we further excluded participants without genotyping information and participants with mismatched sex from genotypic calculations and questionnaire records, leaving 99,920 participants in the GWAS. To maximize the number of recognized COPD patients, we included COPD patients identified in the 1st, 2nd, and 3rd resurvey and those diagnosed in the long-term follow-up (ICD code: J41-J44), resulting in a total of 20,517 COPD patients in the final GWAS. Generalized mixed models were fitted using the R package ‘SAIGE’ (version 1.3.1) to account for sample relatedness, adjusting for age, age square, sex, height, study regions, smoking status, the first ten principal components, and two genotyping arrays. For SNP QC procedure, we first included SNPs with: (1)minor allele frequency (MAF) >0.03, with INFO value >0.3; (2)MAF between 0.01 and 0.03, with INFO value >0.6; (3)MAF between 0.005 and 0.01, with INFO value >0.8; (4)MAF between 0.001 and 0.005, with INFO value >0.9. We then included SNPs that did not deviate from Hardy-Weinberg equilibrium (*P* > 1x10^-6^) and missing rate <5%, resulting in 6,273,713 SNPs left. Clumping procedures were conducted using Plink software (version 1.9), applying the threshold of 0.1 for linkage disequilibrium r-squared, ±500kb for clumping windows, and 5x10^-8^ for significance levels to identify independent lead SNPs.

We next used the independent lead SNPs found in the CKB populations to construct genetic risk scores for MR analyses. F-statistics (above 10) were calculated to assess the associations of GRSs with COPD in the CKB population. Next, we evaluated the associations of IVs with potential confounders and excluded SNPs significantly associated with confounders. The final GRS included in one-sample MR analyses was named ‘CKB GRS 5e8’.

**IVs selection on COPD in the BBJ population**

To try to assess the direction of potential bias, we used GWAS summary statistics from BBJ to construct BBJ-specific GRS for sensitivity analyses using IVs derived only from the BBJ cohort. We first kept SNPs that could be simutaneously found in the BBJ summary statistics and the CKB population. Next, the same SNP QC procedure as what has been done in the CKB population was first applied to retain high quality SNPs, resulting in 5,004,950 SNPs left. Clumping procedures were conducted using Plink software (version 1.9), applying the threshold of 0.1 for linkage disequilibrium r-squared, ±500kb for clumping windows, and 5x10^-8^ for significance levels to identify independent lead SNPs, and 10 lead SNPs were identified. We then evaluated the associations of IVs from BBJ with potential confounders and excluded SNPs significantly associated with confounders. The final GRS included in one-sample MR analyses was named ‘BBJ GRS 5e8’.

Since the inclusion of two heterogeneous SNPs (with opposite effects on COPD between the CKB population and the BBJ population) on chromosome 19 substantially reduced the F-statistics, we additionally constructed a GRS namely ‘BBJ GRS 5e8 nohet’ excluding the two SNPs to address the issue of potential weak instrumental bias, which has made the F statistics increase from 4.7 to 17.0.

**Supplementary Figure 1. The Manhattan plot (left) and Q-Q plots (right) for GWAS results in the CKB population and meta-GWAS results in the CKB and BBJ populations**

The figure on the left shows the Manhattan plot for GWAS results in the CKB population(the inner circle) and meta-GWAS results in the CKB and BBJ populations (the outer circle). Dots plotted in different colors represented the associations' *P* values of SNPs on other chromosomes. The figures on the right show the Q-Q plots for GWAS results in the CKB population(the blue plot) and meta-GWAS results in the CKB and BBJ populations (the yellow circle). The y-axis represented the observed -log_10_(*P*) values of SNPs, and the x-axis represented the expected -log_10_(*P*) of SNPs.

**Supplementary Table 4. The summary information for the SNPs used to construct CKB and meta genetic risk scores**

| SNP | Rsid | CHR | BP | NEA | EA | EAF_CKB | BETA_CKB | P values_CKB | INFO_CKB | GRS used | Direction_Meta | *P* values_Meta |
| --- | --- | --- | --- | --- | --- | --- | --- | --- | --- | --- | --- | --- |
| 6:32128394:C:T | rs9267812 | 6 | 32128394 | C | T | 0.404567 | -0.0750031 | 2.164313e-09 | 0.998768 | CKB GRS 5e8 |  |  |
| 6:32006621:A:C | rs6463 | 6 | 32006621 | A | C | 0.14519 | 0.098825 | 9.246823e-09 | 0.981756 | CKB GRS 5e8 |  |  |
| 1:161500130:A:G^a^ | rs2099684 | 1 | 161500130 | A | G | 0.306585 | 0.0458629 | 0.000418469 | 0.975325 |  | -- | 9.955e-09 |
| 3:25529280:C:T | rs1286664 | 3 | 25529280 | C | T | 0.324289 | -0.0325767 | 0.01135015 | 0.996252 | Meta GRS 5e8 | -- | 8.967e-10 |
| 4:89879196:G:T | rs1585258 | 4 | 89879196 | G | T | 0.594629 | 0.0584009 | 2.472731e-06 | 0.960235 | Meta GRS 5e8 | ++ | 5.536e-13 |
| 4:7850310:T:C | rs61214925 | 4 | 7850310 | T | C | 0.484144 | 0.0482968 | 9.860822e-05 | 0.968867 | Meta GRS 5e8 | -- | 6.101e-10 |
| 6:32124424:G:T^a^ | rs10947233 | 6 | 32124424 | G | T | 0.222462 | -0.0861484 | 3.842103e-09 | 0.999886 |  | -- | 1.459e-12 |
| 6:31239108:T:A | rs1131118 | 6 | 31239108 | T | A | 0.313814 | -0.0449205 | 0.000714064 | 0.977483 | Meta GRS 5e8 | -- | 1.678e-08 |
| 12:112991452:C:T^a^ | rs10850057 | 12 | 112991452 | C | T | 0.85727 | 0.0442908 | 0.01296993 | 0.905556 |  | ++ | 1.478e-08 |
| 12:111414461:T:G^a^ | rs12229654 | 12 | 111414461 | T | G | 0.182912 | 0.0503904 | 0.001227143 | 0.996369 |  | -- | 6.923e-15 |
| 12:112331915:C:T^a^ | rs12231873 | 12 | 112331915 | C | T | 0.592784 | 0.0495954 | 6.85377e-05 | 0.981617 |  | ++ | 2.646e-14 |
| 15:78916187:C:T | rs4887070 | 15 | 78916187 | C | T | 0.518089 | -0.0283973 | 0.02003007 | 0.973045 | Meta GRS 5e8 | -- | 3.155e-09 |

^a^These three SNPs were excluded from constructing Meta GRS 5e8 because they are strongly associated with covariates.

**Supplementary Table 5. The summary information for the SNPs used to construct BBJ genetic risk scores**

| SNP | rsid | CHR | BP | NEA | EA | EAF_BBJ | BETA_BBJ | P values_BBJ | INFO_BBJ | EAF_CKB | BETA_CKB | P values_CKB | INFO_CKB | GRS used |
| --- | --- | --- | --- | --- | --- | --- | --- | --- | --- | --- | --- | --- | --- | --- |
| 3:25529280:C:T | rs1286664 | 3 | 25529280 | C | T | 0.324247867983801 | -0.143897194 | 7.10638162735515e-09 | 0.993287336729195 | 0.324289 | -0.0325767 | 0.01135015 | 0.996252 | BBJ GRS 5e8  & BBJ GRS 5e8 nohet |
| 4:89879196:G:T | rs1585258 | 4 | 89879196 | G | T | 0.655631769717541 | 0.134383115191931 | 4.41766790925497e-08 | 0.993878393207742 | 0.594629 | 0.0584009 | 2.472731e-06 | 0.960235 | BBJ GRS 5e8  & BBJ GRS 5e8 nohet |
| 12:110697330:G:A^a^ | rs117121174 | 12 | 110697330 | G | A | 0.126814204892022 | 0.229595554076806 | 2.19177696014251e-08 | 0.764489444195566 | 0.083901 | 0.0207929 | 0.3483738 | 0.947073 |  |
| 12:111609727:G:T^a^ | rs3809297 | 12 | 111609727 | G | T | 0.23377030106449 | 0.217115084100177 | 2.63221240307463e-14 | 0.940189244301915 | 0.190401 | 0.043447 | 0.005174334 | 0.973325 |  |
| 12:112048359:G:A^a^ | rs7302114 | 12 | 112048359 | G | A | 0.597394040354602 | 0.144878017875602 | 2.33429864393939e-09 | 0.967003111991861 | 0.578677 | 0.0548585 | 1.394072e-05 | 0.964504 |  |
| 12:112645401:G:A^a^ | rs2074356 | 12 | 112645401 | G | A | 0.2180477802207 | 0.261821810406955 | 5.91705606357218e-15 | 0.761539696220688 | 0.141687 | 0.0498088 | 0.003471445 | 0.994088 |  |
| 15:78802869:C:T | rs9788721 | 15 | 78802869 | C | T | 0.674137564307557 | -0.173729763 | 9.11877235762233e-12 | 0.949914319642853 | 0.691612 | -0.00811526 | 0.5351638 | 0.998621 | BBJ GRS 5e8  & BBJ GRS 5e8 nohet |
| 19:41209177:C:T^b^ | rs2288450 | 19 | 41209177 | C | T | 0.163935133062028 | -0.219850423 | 7.25390521496605e-12 | 0.983757914507856 | 0.066729 | 0.0070177 | 0.7744498 | 0.99964 | BBJ GRS 5e8 |
| 19:41324078:G:A^b^ | rs71358934 | 19 | 41324078 | G | A | 0.385242824555326 | 0.14839283490982 | 2.03100079251288e-09 | 0.938317156756422 | 0.349377 | -0.00491307 | 0.7053206 | 0.96138 | BBJ GRS 5e8 |
| 19:41442597:G:A^a^ | rs73038469 | 19 | 41442597 | G | A | 0.174656118642039 | 0.316636368833365 | 1.07904928147649e-20 | 0.857114412197684 | 0.213661 | 0.0076279 | 0.6047702 | 0.995824 |  |

^a^These three SNPs were excluded from constructing BBJ GRS 5e8 & BBJ GRS 5e8 nohet because they are strongly associated with covariates.

^b^These three SNPs were excluded from constructing BBJ GRS 5e8 nohet because they are heterogeneous SNPs in the CKB and BBJ population.

**Supplementary Table 6. The associations between SNPs used to construct genetic risk scores and covariates**

| Covariates | 6:32128394:C:T | 6:32006621:A:C | 1:161500130:A:G | 3:25529280:C:T | 4:89879196:G:T | 4:7850310:T:C | 6:32124424:G:T | 6:31239108:T:A | 12:112991452:C:T | 12:111414461:T:G | 12:112331915:C:T | 15:78916187:C:T |
| --- | --- | --- | --- | --- | --- | --- | --- | --- | --- | --- | --- | --- |
| Education | 0.884 | 0.488 | 0.970 | 0.36 | 0.554 | 0.211 | 0.568 | 0.351 | 0.989 | 0.637 | 0.085 | 0.928 |
| Occupation | 0.884 | 0.524 | 0.970 | 0.485 | 0.936 | 0.932 | 0.863 | 0.763 | 0.461 | 0.045# | 0.216 | 0.928 |
| Income | 0.608 | 0.905 | 0.979 | 0.513 | 0.936 | 0.675 | 0.568 | 0.676 | 0.167 | 0.965 | 0.399 | 0.785 |
| Marital status | 0.884 | 0.856 | 0.970 | 0.828 | 0.936 | 0.765 | 0.568 | 0.846 | 0.989 | 0.966 | 0.216 | 0.956 |
| Smoking | 0.884 | 0.861 | 0.970 | 0.828 | 0.554 | 0.940 | 0.261 | 0.368 | 0.989 | 0.405 | 0.216 | 0.337 |
| Alcohol drinking | 0.884 | 0.524 | 0.970 | 0.754 | 0.936 | 0.765 | 0.568 | 0.715 | 0.000# | 0.000# | 0.000# | 0.928 |
| Physical activity | 0.884 | 0.296 | 0.970 | 0.828 | 0.984 | 0.765 | 0.568 | 0.403 | 0.989 | 0.965 | 0.555 | 0.928 |
| Fruit intake | 0.884 | 0.811 | 0.970 | 0.828 | 0.936 | 0.940 | 0.568 | 0.875 | 0.254 | 0.000# | 0.085 | 0.928 |
| Vegetable intake | 0.884 | 0.951 | 0.970 | 0.360 | 0.936 | 0.765 | 0.568 | 0.763 | 0.167 | 0.743 | 0.148 | 0.785 |
| Meat intake | 0.884 | 0.861 | 0.970 | 0.828 | 0.554 | 0.765 | 0.550 | 0.722 | 0.989 | 0.346 | 0.676 | 0.928 |
| Passive smoking | 0.884 | 0.524 | 0.970 | 0.754 | 0.984 | 0.940 | 0.863 | 0.846 | 0.989 | 0.677 | 0.148 | 0.928 |
| Primary cook fuel | 0.884 | 0.861 | 0.863 | 0.978 | 0.936 | 0.713 | 0.872 | 0.676 | 0.989 | 0.656 | 0.292 | 0.928 |
| Primary heating fuel | 0.884 | 0.543 | 0.970 | 0.754 | 0.936 | 0.940 | 0.568 | 0.763 | 0.989 | 0.091 | 0.423 | 0.928 |
| BMI | 0.884 | 0.296 | 0.863 | 0.754 | 0.554 | 0.983 | 0.907 | 0.633 | 0.030# | 0.035# | 0.085 | 0.928 |
| Abdominal obesity | 0.884 | 0.524 | 0.863 | 0.754 | 0.936 | 0.940 | 0.550 | 0.631 | 0.030# | 0.000# | 0.001# | 0.956 |
| Self-rated health | 0.884 | 0.524 | 0.047# | 0.976 | 0.554 | 0.765 | 0.863 | 0.763 | 0.989 | 0.239 | 0.338 | 0.981 |
| Hypertension | 0.105 | 0.373 | 0.970 | 0.360 | 0.554 | 0.940 | 0.001# | 0.143 | 0.989 | 0.000# | 0.148 | 0.928 |
| Diabetes | 0.105 | 0.861 | 0.970 | 0.828 | 0.554 | 0.963 | 0.490 | 0.423 | 0.566 | 0.759 | 0.148 | 0.785 |

Each column represents the false discovery rate-corrected p-value for the association between a single SNP and each of the examined covariates.

**P*<0.05; #*False discovery rate*<0.05*.*

**Supplementary Table 6 extended. The associations between SNPs used to construct genetic risk scores and covariates**

| Covariates | 3:25529280:C:T | 4:89879196:G:T | 12:110697330:G:A | 12:111609727:G:T | 12.112048359.G.A_A..G. | 12:112645401:G:A | 15:78802869:C:T | 19:41209177:C:T | 19:41324078:G:A | 19:41442597:G:A |
| --- | --- | --- | --- | --- | --- | --- | --- | --- | --- | --- |
| Education | 0.36 | 0.99 | 0.938 | 0.998 | 0.438 | 0.922 | 0.926 | 0.98 | 0.921 | 0.989 |
| Occupation | 0.485 | 0.99 | 0.416 | 0.085 | 0.152 | 0.061 | 0.926 | 0.98 | 0.921 | 0.989 |
| Income | 0.513 | 0.99 | 0.915 | 0.998 | 0.518 | 0.828 | 0.926 | 0.98 | 0.921 | 0.989 |
| Marital status | 0.828 | 0.99 | 0.452 | 0.998 | 0.521 | 0.828 | 0.926 | 0.98 | 0.921 | 0.989 |
| Smoking | 0.828 | 0.99 | 0.622 | 0.393 | 0.881 | 0.488 | 0.07 | 0.98 | 0.963 | 0.989 |
| Alcohol drinking | 0.754 | 0.99 | 0.000# | 0.000# | 0.000# | 0.000# | 0.073 | 0.98 | 0.921 | 0.193 |
| Physical activity | 0.828 | 0.99 | 0.938 | 0.998 | 0.881 | 0.794 | 0.926 | 0.98 | 0.921 | 0.989 |
| Fruit intake | 0.828 | 0.99 | 0.000# | 0.000# | 0.108 | 0.000# | 0.926 | 0.996 | 0.921 | 0.989 |
| Vegetable intake | 0.36 | 0.99 | 0.772 | 0.626 | 0.438 | 0.828 | 0.926 | 0.98 | 0.921 | 0.989 |
| Meat intake | 0.828 | 0.721 | 0.283 | 0.39 | 0.93 | 0.494 | 0.926 | 0.98 | 0.963 | 0.989 |
| Passive smoking | 0.754 | 0.99 | 0.679 | 0.869 | 0.126 | 0.984 | 0.926 | 0.98 | 0.921 | 0.193 |
| Primary cook fuel | 0.978 | 0.99 | 0.114 | 0.39 | 0.181 | 0.645 | 0.926 | 0.98 | 0.921 | 0.989 |
| Primary heating fuel | 0.754 | 0.99 | 0.041# | 0.063 | 0.881 | 0.005# | 0.926 | 0.98 | 0.921 | 0.989 |
| BMI | 0.754 | 0.99 | 0.851 | 0.106 | 0.053 | 0.011# | 0.926 | 0.98 | 0.921 | 0.989 |
| Abdominal obesity | 0.754 | 0.99 | 0.114 | 0.000# | 0.003# | 0.000# | 0.926 | 0.98 | 0.921 | 0.989 |
| Self-rated health | 0.976 | 0.721 | 0.847 | 0.372 | 0.654 | 0.828 | 0.926 | 0.98 | 0.921 | 0.989 |
| Hypertension | 0.36 | 0.721 | 0.316 | 0.000# | 0.152 | 0.000# | 0.926 | 0.98 | 0.921 | 0.989 |
| Diabetes | 0.828 | 0.721 | 0.932 | 0.67 | 0.181 | 0.828 | 0.926 | 0.98 | 0.921 | 0.989 |

Each column represents the false discovery rate-corrected p-value for the association between a single SNP and each of the examined covariates.

**P*<0.05; #*False discovery rate*<0.05*.*

**Supplementary Table 7. The associations between constructed genetic risk scores and covariates**

| Covariates | CKB GRS 5e8 | Meta GRS 5e8 | BBJ GRS 5e8 | BBJ GRS 5e8 nohet |
| --- | --- | --- | --- | --- |
| Education | 0.482 | 0.586 | 0.744 | 0.403 |
| Occupation | 0.987 | 0.849 | 0.744 | 0.713 |
| Income | 0.707 | 0.829 | 0.948 | 0.713 |
| Marital status | 0.987 | 0.993 | 0.744 | 0.775 |
| Smoking | 0.987 | 0.452 | 0.744 | 0.403 |
| Alcohol drinking | 0.984 | 0.893 | 0.744 | 0.403 |
| Physical activity | 0.984 | 0.753 | 0.948 | 0.984 |
| Fruit intake | 0.984 | 0.849 | 0.744 | 0.73 |
| Vegetable intake | 0.987 | 0.753 | 0.744 | 0.713 |
| Meat intake | 0.984 | 0.756 | 0.744 | 0.713 |
| Passive smoking | 0.984 | 0.648 | 0.744 | 0.713 |
| Primary cook fuel | 0.987 | 0.586 | 0.744 | 0.713 |
| Primary heating fuel | 0.987 | 0.893 | 0.744 | 0.775 |
| BMI | 0.482 | 0.893 | 0.744 | 0.775 |
| Abdominal obesity | 0.899 | 0.586 | 0.744 | 0.713 |
| Self-rated health | 0.687 | 0.753 | 0.744 | 0.403 |
| Hypertension | 0.055 | 0.586 | 0.783 | 0.822 |
| Diabetes | 0.482 | 0.753 | 0.783 | 0.713 |

Each column represents the false discovery rate-corrected p-value for the association between the constructed GRSs and each of the examined covariates.

**P*<0.05; #*False discovery rate*<0.05*.*

**Supplementary Table 8.** **Associations of prevalent COPD at baseline with** **selected digestive diseases**

| Diseases | No.event | HR(95% CI) | pval | fdrp |
| --- | --- | --- | --- | --- |
| Digestive diseases | 112,392 | 1.05(1.03-1.08)# | <0.001 | <0.001 |
| Diseases of esophagus | 2,750 | 0.99(0.86-1.14) | 0.902 | 0.998 |
| Gastro-esophageal reflux disease [GERD] | 2,080 | 0.98(0.83-1.16) | 0.819 | 0.998 |
| Peptic ulcer | 7,156 | 1.07(0.99-1.16) | 0.086 | 0.280 |
| Gastric ulcer | 4,391 | 1.10(1.00-1.22) | 0.054 | 0.210 |
| Gastrointestinal obstruction | 5,710 | 1.08(0.99-1.17) | 0.099 | 0.280 |
| Vascular disorders of intestine | 237 | 0.66(0.37-1.17) | 0.153 | 0.342 |
| Gastrointestinal inflammation | 47,585 | 1.11(1.07-1.15)# | <0.001 | <0.001 |
| Inflammatory bowel disease | 539 | 1.19(0.86-1.66) | 0.291 | 0.430 |
| Ulceration of the lower GI tract | 115 | 0.99(0.50-1.93) | 0.968 | 0.998 |
| Duodenitis | 259 | 0.64(0.37-1.09) | 0.099 | 0.280 |
| Gastritis | 44,794 | 1.11(1.07-1.14)# | <0.001 | <0.001 |
| Functional intestinal disorder | 631 | 1.02(0.78-1.33) | 0.898 | 0.998 |
| Irritable bowel syndrome | 172 | 1.14(0.64-2.01) | 0.657 | 0.926 |
| Intestinal infection | 18,301 | 1.00(0.95-1.05) | 0.977 | 0.998 |
| Abdominal pain | 9,077 | 1.20(1.12-1.28)# | <0.001 | <0.001 |
| Functional dyspepsia | 9,077 | 1.20(1.12-1.28)# | <0.001 | <0.001 |
| Intestinal or peritoneal adhesions | 901 | 0.87(0.68-1.10) | 0.232 | 0.359 |
| Abnormality of the peritoneum | 2,938 | 0.92(0.80-1.05) | 0.215 | 0.358 |
| Peritonitis | 2,850 | 0.91(0.79-1.05) | 0.190 | 0.353 |
| Hepatitis | 636 | 0.79(0.55-1.12) | 0.178 | 0.353 |
| Chronic hepatitis | 545 | 0.73(0.49-1.08) | 0.118 | 0.306 |
| Chronic liver disease | 7,855 | 1.01(0.93-1.10) | 0.754 | 0.998 |
| Fibrosis and cirrhosis of liver | 3,441 | 0.99(0.87-1.12) | 0.850 | 0.998 |
| Fatty liver disease (FLD) | 2,959 | 0.99(0.86-1.14) | 0.849 | 0.998 |
| Hepatic failure | 544 | 1.21(0.91-1.63) | 0.193 | 0.353 |
| Chronic nonalcoholic liver disease | 2,631 | 0.91(0.78-1.06) | 0.219 | 0.358 |
| Disorders of the gallbladder | 24,861 | 1.06(1.01-1.11)* | 0.020 | 0.089 |
| Gallstones [Cholelithiasis] | 14,725 | 1.00(0.94-1.06) | 0.998 | 0.998 |
| Cholecystitis | 11,387 | 1.13(1.05-1.20)# | <0.001 | 0.003 |
| Gastrointestinal hemorrhage | 9,182 | 1.05(0.98-1.13) | 0.155 | 0.342 |

HRs and 95% CIs were calculated using stratified Cox regression models, stratified by age (in 5-year intervals), sex and living areas, adjusting for education, household income, occupation, marital status, alcohol consumption, smoking status, passive smoking status, physical activity levels, primary cooking fuel, primary heating fuel usage, and consumption frequency of fresh fruits, fresh vegetables, meat, general obesity and abdominal obesity, as well as self-rated health status, prevalent hypertension and prevalent diabetes at baseline.

*P<0.05; #False discovery rate<0.05.

Abbreviations: COPD, chronic obstructive pulmonary disease; HR, hazard ratio; CI, confidence interval.

**Supplementary Table 9.** **Associations of prevalent COPD at baseline with the frequency of occurrence for the selected digestive diseases**

| Diseases | Non-COPD participants | COPD patients | |
| --- | --- | --- | --- |
|  |  | IRR (95% CI) | P values |
| Digestive diseases | 1.00 | 1.02(0.99-1.06) | 0.136 |
| Diseases of esophagus | 1.00 | 0.95(0.78-1.16) | 0.617 |
| Gastro-esophageal reflux disease [GERD] | 1.00 | 0.94(0.79-1.13) | 0.535 |
| Peptic ulcer | 1.00 | 1.01(0.90-1.14) | 0.846 |
| Gastric ulcer | 1.00 | 1.03(0.92-1.15) | 0.599 |
| Gastrointestinal obstruction | 1.00 | 1.01(0.88-1.16) | 0.913 |
| Vascular disorders of intestine | 1.00 | 0.54(0.29-1.01) | 0.055 |
| Gastrointestinal inflammation | 1.00 | 1.07(1.02-1.13) | 0.004* |
| Inflammatory bowel disease | 1.00 | 1.58(0.96-2.57) | 0.069 |
| Ulceration of the lower GI tract | 1.00 | 0.73(0.24-2.19) | 0.576 |
| Duodenitis | 1.00 | 0.98(0.39-2.50) | 0.970 |
| Gastritis | 1.00 | 1.06(1.02-1.11) | 0.003* |
| Functional intestinal disorder | 1.00 | 0.91(0.65-1.27) | 0.572 |
| Irritable bowel syndrome | 1.00 | 1.28(0.49-3.33) | 0.609 |
| Intestinal infection | 1.00 | 0.98(0.93-1.04) | 0.525 |
| Abdominal pain | 1.00 | 1.07(0.50-2.26) | 0.865 |
| Functional dyspepsia | 1.00 | 1.06(0.49-2.29) | 0.889 |
| Intestinal or peritoneal adhesions | 1.00 | 0.85(0.66-1.09) | 0.191 |
| Abnormality of the peritoneum | 1.00 | 0.92(0.77-1.11) | 0.396 |
| Peritonitis | 1.00 | 0.88(0.76-1.01) | 0.065 |
| Hepatitis | 1.00 | 0.61(0.38-0.97) | 0.039* |
| Chronic hepatitis | 1.00 | 0.55(0.34-0.89) | 0.016* |
| Chronic liver disease | 1.00 | 0.88(0.76-1.01) | 0.075 |
| Fibrosis and cirrhosis of liver | 1.00 | 0.81(0.66-1.00) | 0.054 |
| Fatty liver disease (FLD) | 1.00 | 1.09(0.95-1.27) | 0.226 |
| Hepatic failure | 1.00 | 1.08(0.77-1.53) | 0.642 |
| Chronic non-alcoholic liver disease | 1.00 | 0.78(0.63-0.96) | 0.020* |
| Disorders of the gallbladder | 1.00 | 1.02(0.95-1.09) | 0.612 |
| Gallstones [Cholelithiasis] | 1.00 | 0.96(0.90-1.03) | 0.298 |
| Cholecystitis | 1.00 | 1.10(1.01-1.20) | 0.027* |
| Gastrointestinal hemorrhage | 1.00 | 0.97(0.89-1.06) | 0.532 |

IRRs and 95% CIs were calculated using negative , stratified by age (in 5-year intervals), sex and living areas, adjusting for education, household income, occupation, marital status, alcohol consumption, smoking status, passive smoking status, physical activity levels, primary cooking fuel, primary heating fuel usage, and consumption frequency of fresh fruits, fresh vegetables, meat, general obesity and abdominal obesity, as well as self-rated health status, prevalent hypertension and prevalent diabetes at baseline.

**P*<0.05; #*False discovery rate*<0.05*.*

Abbreviations: COPD, chronic obstructive pulmonary disease; IRR, incidence rate ratio; CI, confidence interval

**Supplementary Figure 2.** **Associations of prevalent COPD at baseline with selected digestive diseases in old (**≥**60 years old) and young (**<**60 years old) participants**

The centers of the squares represent the point estimates for HRs, and the edges represent the lower and upper bounds for 95% CIs. The sizes of the squares are directly proportional to the reciprocals of the estimated standard errors of the effect sizes. HRs and 95% CIs were calculated using stratified Cox regression models, stratified by age (in 5-year intervals), sex and living areas, adjusting for education, household income, occupation, marital status, alcohol consumption, smoking status, passive smoking status, physical activity levels, primary cooking fuel, primary heating fuel usage, and consumption frequency of fresh fruits, fresh vegetables, meat, general obesity and abdominal obesity, as well as self-rated health status, prevalent hypertension and prevalent diabetes at baseline. *P* values for interactions of COPD with these factors were obtained from likelihood-ratio tests of the two models before and after adding the interaction term.

**P*<0.05; #*False discovery rate*<0.05*.*

Abbreviations: COPD, chronic obstructive pulmonary disease; HR, hazard ratio; CI, confidence interval.

**Supplementary Figure 3. Associations of prevalent COPD at baseline with selected digestive diseases in male and female participants**

The centers of the squares represent the point estimates for HRs, and the edges represent the lower and upper bounds for 95% CIs. The sizes of the squares are directly proportional to the reciprocals of the estimated standard errors of the effect sizes. HRs and 95% CIs were calculated using stratified Cox regression models, stratified by age (in 5-year intervals), and living areas, adjusting for education, household income, occupation, marital status, alcohol consumption, smoking status, passive smoking status, physical activity levels, primary cooking fuel, primary heating fuel usage, and consumption frequency of fresh fruits, fresh vegetables, meat, general obesity and abdominal obesity, as well as self-rated health status, prevalent hypertension and prevalent diabetes at baseline. *P* values for interactions of COPD with these factors were obtained from likelihood-ratio tests of the two models before and after adding the interaction term.

**P*<0.05; #*False discovery rate*<0.05*.*

Abbreviations: COPD, chronic obstructive pulmonary disease; HR, hazard ratio; CI, confidence interval.

**Supplementary Figure 4. Associations of prevalent COPD at baseline with selected digestive diseases in low BMI (BMIl, <24kg/****m^2^) and high BMI (BMIh, ≥24kg/m^2^) participants**

The centers of the squares represent the point estimates for HRs, and the edges represent the lower and upper bounds for 95% CIs. The sizes of the squares are directly proportional to the reciprocals of the estimated standard errors of the effect sizes. HRs and 95% CIs were calculated using stratified Cox regression models, stratified by age (in 5-year intervals), sex and living areas, adjusting for education, household income, occupation, marital status, alcohol consumption, smoking status, passive smoking status, physical activity levels, primary cooking fuel, primary heating fuel usage, and consumption frequency of fresh fruits, fresh vegetables, meat, and abdominal obesity, as well as self-rated health status, prevalent hypertension and prevalent diabetes at baseline. *P* values for interactions of COPD with these factors were obtained from likelihood-ratio tests of the two models before and after adding the interaction term.

**P*<0.05; #*False discovery rate*<0.05*.*

Abbreviations: COPD, chronic obstructive pulmonary disease; HR, hazard ratio; CI, confidence interval. BMI, body mass index.

**Supplementary Figure 5. Associations of prevalent COPD at baseline with selected digestive diseases in never-smokers and ever-smokers**

The centers of the squares represent the point estimates for HRs, and the edges represent the lower and upper bounds for 95% CIs. The sizes of the squares are directly proportional to the reciprocals of the estimated standard errors of the effect sizes. HRs and 95% CIs were calculated using stratified Cox regression models, stratified by age (in 5-year intervals), sex and living areas, adjusting for education, household income, occupation, marital status, alcohol consumption, passive smoking status, physical activity levels, primary cooking fuel, primary heating fuel usage, and consumption frequency of fresh fruits, fresh vegetables, meat, general obesity and abdominal obesity, as well as self-rated health status, prevalent hypertension and prevalent diabetes at baseline. *P* values for interactions of COPD with these factors were obtained from likelihood-ratio tests of the two models before and after adding the interaction term.

**P*<0.05; #*False discovery rate*<0.05*.*

Abbreviations: COPD, chronic obstructive pulmonary disease; HR, hazard ratio; CI, confidence interval.

**Supplementary Figure 6. Associations of prevalent COPD at baseline with selected digestive diseases in non-daily drinkers and daily drinkers**

The centers of the squares represent the point estimates for HRs, and the edges represent the lower and upper bounds for 95% CIs. The sizes of the squares are directly proportional to the reciprocals of the estimated standard errors of the effect sizes. HRs and 95% CIs were calculated using stratified Cox regression models, stratified by age (in 5-year intervals), sex and living areas, adjusting for education, household income, occupation, marital status, smoking status, passive smoking status, physical activity levels, primary cooking fuel, primary heating fuel usage, and consumption frequency of fresh fruits, fresh vegetables, meat, general obesity and abdominal obesity, as well as self-rated health status, prevalent hypertension and prevalent diabetes at baseline. *P* values for interactions of COPD with these factors were obtained from likelihood-ratio tests of the two models before and after adding the interaction term.

**P*<0.05; #*False discovery rate*<0.05.

Abbreviations: COPD, chronic obstructive pulmonary disease; HR, hazard ratio; CI, confidence interval.

**Supplementary Table 10. The detailed information on the incident densities of selected digestive diseases**

| Disease | Non-COPD participants  with FEV1≥ 80% predicted | Non-COPD participants  at PRISm stage | COPD patients at GOLD stage 1 | COPD patients at GOLD stage 2 | COPD patients at GOLD stage 3 | COPD patients at GOLD stage 4 |
| --- | --- | --- | --- | --- | --- | --- |
| GI_ Digestive diseases | 17.66 | 20.21 | 20.3 | 23.67 | 30.74 | 37.88 |
| GI_510 Diseases of the esophagus | 0.35 | 0.35 | 0.46 | 0.43 | 0.51 | 0.50 |
| GI_511 Gastroesophageal reflux disease [GERD] | 0.27 | 0.25 | 0.35 | 0.26 | 0.37 | 0.28 |
| GI_513 Peptic ulcer | 0.89 | 1.06 | 1.12 | 1.48 | 2.07 | 2.09 |
| GI_513.2 Gastric ulcer | 0.53 | 0.68 | 0.74 | 0.96 | 1.57 | 1.33 |
| GI_514 Gastrointestinal obstruction | 0.66 | 0.86 | 1.01 | 1.19 | 1.48 | 2.18 |
| GI_517 Vascular disorders of the intestine | 0.03 | 0.03 | 0.02 | 0.02 | 0.03 | 0.06 |
| GI_522 Gastrointestinal inflammation | 6.10 | 7.02 | 7.37 | 8.63 | 12.05 | 13.86 |
| GI_522.1 Inflammatory bowel disease | 0.07 | 0.08 | 0.06 | 0.10 | 0.08 | 0.06 |
| GI_522.7 Ulceration of the lower GI tract | 0.02 | 0.01 | 0.03 | 0.02 | 0 | 0 |
| GI_522.8 Duodenitis | 0.03 | 0.03 | 0.03 | 0.03 | 0.05 | 0 |
| GI_522.9 Gastritis | 5.71 | 6.63 | 6.93 | 8.07 | 11.47 | 13.66 |
| GI_524 Functional intestinal disorder | 0.08 | 0.07 | 0.15 | 0.08 | 0.18 | 0.33 |
| GI_524.1 Irritable bowel syndrome | 0.02 | 0.03 | 0.04 | 0.02 | 0.02 | 0 |
| GI_526 Intestinal infection | 2.36 | 2.31 | 2.86 | 3.24 | 4.58 | 4.57 |
| GI_527 Abdominal pain | 1.08 | 1.28 | 1.21 | 2.07 | 3.21 | 5.13 |
| GI_527.3 Functional dyspepsia | 1.08 | 1.28 | 1.21 | 2.07 | 3.21 | 5.13 |
| GI_535 Intestinal or peritoneal adhesions | 0.11 | 0.13 | 0.16 | 0.15 | 0.22 | 0 |
| GI_537 Abnormality of the peritoneum | 0.37 | 0.39 | 0.46 | 0.45 | 0.60 | 0.56 |
| GI_537.1 Peritonitis | 0.36 | 0.38 | 0.46 | 0.43 | 0.55 | 0.56 |
| GI_540 Hepatitis | 0.08 | 0.10 | 0.04 | 0.06 | 0.15 | 0.11 |
| GI_540.1 Chronic hepatitis | 0.07 | 0.09 | 0.03 | 0.04 | 0.13 | 0.11 |
| GI_542 Chronic liver disease | 0.96 | 1.18 | 0.97 | 1.24 | 1.66 | 2.88 |
| GI_542.1 Fibrosis and cirrhosis of the liver | 0.40 | 0.57 | 0.43 | 0.58 | 0.84 | 1.40 |
| GI_542.2 Fatty liver disease (FLD) | 0.37 | 0.42 | 0.35 | 0.43 | 0.57 | 0.90 |
| GI_542.3 Hepatic failure | 0.07 | 0.08 | 0.07 | 0.13 | 0.09 | 0.28 |
| GI_542.7 Chronic nonalcoholic liver disease | 0.31 | 0.43 | 0.30 | 0.39 | 0.50 | 0.79 |
| GI_550 Disorders of the gallbladder | 3.16 | 4.33 | 3.53 | 4.47 | 5.57 | 7.38 |
| GI_550.1 Gallstones [Cholelithiasis] | 1.90 | 2.40 | 2.19 | 2.45 | 3.02 | 4.09 |
| GI_550.2 Cholecystitis | 1.36 | 2.14 | 1.43 | 2.21 | 2.87 | 3.93 |
| GI_557 Gastrointestinal hemorrhage | 1.10 | 1.29 | 1.42 | 1.91 | 2.45 | 3.14 |

Incident densities referred to the number of cases that occurred during follow-up per 1,000 person-years.

Abbreviations: COPD, chronic obstructive pulmonary disease. PRISm, preserved ratio impaired spirometry.

**Supplementary Table 11. Associations of airflow obstruction severity in COPD at baseline with selected digestive diseases**

| Disease | Non-COPD participants  with FEV1≥ 80% predicted | Non-COPD participants  at PRISm stage | COPD patients at GOLD stage 1 | COPD patients at GOLD stage 2 | COPD patients at GOLD stage 3 | COPD patients at GOLD stage 4 | *P* for trend |
| --- | --- | --- | --- | --- | --- | --- | --- |
| GI_ Digestive diseases | 1.00 | 1.05 (1.03 - 1.06)# | 1.05 (1.01 - 1.10) | 1.04 (1.01 - 1.08)# | 1.10 (1.05 - 1.16)# | 1.30 (1.19 - 1.42)# | <0.001# |
| GI_510 Diseases of the esophagus | 1.00 | 1.02 (0.93 - 1.12) | 1.16 (0.91 - 1.47) | 0.92 (0.75 - 1.12) | 0.98 (0.72 - 1.33) | 1.00 (0.52 - 1.93) | 0.909 |
| GI_511 Gastroesophageal reflux disease [GERD] | 1.00 | 0.93 (0.83 - 1.04) | 1.21 (0.92 - 1.58) | 0.80 (0.62 - 1.03) | 1.05 (0.73 - 1.50) | 0.87 (0.36 - 2.10) | 0.289 |
| GI_513 Peptic ulcer | 1.00 | 1.00 (0.94 - 1.06) | 1.00 (0.86 - 1.17) | 1.06 (0.94 - 1.18) | 1.19 (1.01 - 1.39) | 1.13 (0.81 - 1.58) | 0.067 |
| GI_513.2 Gastric ulcer | 1.00 | 0.98 (0.91 - 1.05) | 1.05 (0.86 - 1.28) | 1.03 (0.90 - 1.19) | 1.31 (1.09 - 1.57)# | 1.02 (0.68 - 1.55) | 0.077 |
| GI_514 Gastrointestinal obstruction | 1.00 | 1.19 (1.11 - 1.26)# | 1.14 (0.97 - 1.34) | 1.10 (0.97 - 1.24) | 1.11 (0.93 - 1.33) | 1.60 (1.17 - 2.21)# | <0.001# |
| GI_517 Vascular disorders of the intestine | 1.00 | 1.00 (0.73 - 1.39) | 0.53 (0.17 - 1.66) | 0.63 (0.28 - 1.44) | 0.80 (0.25 - 2.54) | 1.36 (0.19 - 9.85) | 0.300 |
| GI_522 Gastrointestinal inflammation | 1.00 | 1.01 (0.99 - 1.04) | 1.14 (1.08 - 1.22)# | 1.08 (1.03 - 1.13)# | 1.14 (1.07 - 1.22)# | 1.18 (1.03 - 1.34)# | <0.001# |
| GI_522.1 Inflammatory bowel disease | 1.00 | 0.93 (0.76 - 1.15) | 1.04 (0.55 - 1.97) | 1.36 (0.89 - 2.08) | 0.98 (0.46 - 2.10) | 0.59 (0.08 - 4.28) | 0.679 |
| GI_522.7 Ulceration of the lower GI tract | 1.00 | 0.99 (0.61 - 1.60) | 1.31 (0.48 - 3.62) | 1.22 (0.52 - 2.88) | 0.00 (0.00 - Inf) | 0.00 (0.00 - Inf) | 0.660 |
| GI_522.8 Duodenitis | 1.00 | 0.84 (0.61 - 1.15) | 0.62 (0.23 - 1.68) | 0.54 (0.25 - 1.17) | 0.90 (0.33 - 2.48) | 0.00 (0.00 - Inf) | 0.056 |
| GI_522.9 Gastritis | 1.00 | 1.02 (0.99 - 1.04) | 1.15 (1.08 - 1.22)# | 1.07 (1.02 - 1.12)# | 1.15 (1.07 - 1.23)# | 1.23 (1.08 - 1.40)# | <0.001# |
| GI_524 Functional intestinal disorder | 1.00 | 1.01 (0.82 - 1.24) | 1.15 (0.75 - 1.76) | 0.66 (0.42 - 1.05) | 1.46 (0.87 - 2.44) | 2.56 (1.13 - 5.81) | 0.582 |
| GI_524.1 Irritable bowel syndrome | 1.00 | 1.10 (0.76 - 1.59) | 2.04 (0.94 - 4.42) | 0.83 (0.34 - 2.07) | 0.90 (0.22 - 3.74) | 0.00 (0.00 - Inf) | 0.842 |
| GI_526 Intestinal infection | 1.00 | 1.02 (0.98 - 1.06) | 1.07 (0.97 - 1.18) | 0.97 (0.90 - 1.04) | 1.03 (0.93 - 1.14) | 0.94 (0.75 - 1.17) | 0.820 |
| GI_527 Abdominal pain | 1.00 | 1.07 (1.02 - 1.13)# | 1.01 (0.87 - 1.17) | 1.22 (1.11 - 1.34)# | 1.30 (1.15 - 1.48)# | 1.78 (1.44 - 2.20)# | <0.001# |
| GI_527.3 Functional dyspepsia | 1.00 | 1.07 (1.02 - 1.13)# | 1.01 (0.87 - 1.17) | 1.22 (1.11 - 1.34)# | 1.30 (1.15 - 1.48)# | 1.78 (1.44 - 2.20)# | <0.001# |
| GI_535 Intestinal or peritoneal adhesions | 1.00 | 1.24 (1.06 - 1.45)# | 1.13 (0.76 - 1.70) | 0.85 (0.61 - 1.18) | 1.05 (0.66 - 1.67) | 0.00 (0.00 - Inf) | 0.820 |
| GI_537 Abnormality of the peritoneum | 1.00 | 1.05 (0.96 - 1.15) | 1.04 (0.82 - 1.32) | 0.85 (0.70 - 1.04) | 0.98 (0.74 - 1.30) | 0.92 (0.49 - 1.71) | 0.504 |
| GI_537.1 Peritonitis | 1.00 | 1.04 (0.95 - 1.14) | 1.07 (0.84 - 1.36) | 0.84 (0.69 - 1.03) | 0.92 (0.69 - 1.24) | 0.93 (0.50 - 1.74) | 0.343 |
| GI_540 Hepatitis | 1.00 | 1.16 (0.97 - 1.40) | 0.52 (0.23 - 1.18) | 0.67 (0.39 - 1.15) | 1.61 (0.91 - 2.84) | 1.17 (0.29 - 4.74) | 0.818 |
| GI_540.1 Chronic hepatitis | 1.00 | 1.16 (0.95 - 1.42) | 0.51 (0.21 - 1.24) | 0.56 (0.30 - 1.06) | 1.56 (0.84 - 2.89) | 1.30 (0.32 - 5.27) | 0.988 |
| GI_542 Chronic liver disease | 1.00 | 1.13 (1.07 - 1.19)# | 0.94 (0.80 - 1.11) | 0.99 (0.88 - 1.11) | 1.17 (0.99 - 1.39) | 2.07 (1.57 - 2.74)# | <0.001# |
| GI_542.1 Fibrosis and cirrhosis of the liver | 1.00 | 1.11 (1.02 - 1.20)# | 0.91 (0.71 - 1.16) | 0.95 (0.80 - 1.13) | 1.16 (0.91 - 1.47) | 1.98 (1.33 - 2.96)# | 0.076 |
| GI_542.2 Fatty liver disease (FLD) | 1.00 | 1.18 (1.08 - 1.28)# | 0.93 (0.71 - 1.23) | 0.95 (0.78 - 1.16) | 1.21 (0.91 - 1.62) | 2.05 (1.25 - 3.37)# | 0.027 |
| GI_542.3 Hepatic failure | 1.00 | 1.11 (0.90 - 1.37) | 0.82 (0.44 - 1.55) | 1.43 (0.98 - 2.08) | 1.05 (0.51 - 2.14) | 3.59 (1.46 - 8.83)# | 0.039 |
| GI_542.7 Chronic nonalcoholic liver disease | 1.00 | 1.12 (1.02 - 1.22)# | 0.86 (0.64 - 1.15) | 0.90 (0.73 - 1.11) | 1.02 (0.75 - 1.39) | 1.69 (0.99 - 2.88) | 0.586 |
| GI_550 Disorders of the gallbladder | 1.00 | 1.09 (1.05 - 1.12)# | 1.00 (0.91 - 1.09) | 1.06 (0.99 - 1.13) | 1.18 (1.07 - 1.30)# | 1.62 (1.35 - 1.94)# | <0.001# |
| GI_550.1 Gallstones [Cholelithiasis] | 1.00 | 1.12 (1.08 - 1.17)# | 0.99 (0.89 - 1.12) | 0.99 (0.90 - 1.07) | 1.12 (0.98 - 1.27) | 1.62 (1.27 - 2.06)# | <0.001# |
| GI_550.2 Cholecystitis | 1.00 | 1.07 (1.03 - 1.12)# | 0.99 (0.86 - 1.14) | 1.15 (1.05 - 1.26)# | 1.24 (1.08 - 1.42)# | 1.67 (1.30 - 2.14)# | <0.001# |
| GI_557 Gastrointestinal hemorrhage | 1.00 | 1.06 (1.01 - 1.11) | 0.95 (0.83 - 1.09) | 1.07 (0.98 - 1.18) | 1.15 (1.00 - 1.32) | 1.44 (1.10 - 1.87)# | 0.003# |

HRs and 95% CIs were calculated using stratified Cox regression models, stratified by age (in 5-year intervals), sex and living areas, adjusting for education, household income, occupation, marital status, alcohol consumption, smoking status, passive smoking status, physical activity levels, primary cooking fuel, primary heating fuel usage, and consumption frequency of fresh fruits, fresh vegetables, meat, general obesity and abdominal obesity, as well as self-rated health status, prevalent hypertension and prevalent diabetes at baseline.

\#*False discovery rate*<0.05.

Abbreviations: COPD, chronic obstructive pulmonary disease. PRISm, preserved ratio impaired spirometry.

**Supplementary Table 12. Associations of airflow obstruction severity in COPD at baseline with selected digestive diseases in never-smokers and ever-smokers**

| Disease | Subgroup | Non-COPD participants  with FEV1≥ 80% predicted | Non-COPD participants  at PRISm stage | COPD patients at GOLD stage 1 | COPD patients at GOLD stage 2 | COPD patients at GOLD stage 3 | COPD patients at GOLD stage 4 | p for interaction |
| --- | --- | --- | --- | --- | --- | --- | --- | --- |
| GI_ Digestive diseases | Never-smoker | 1.00 | 1.05 (1.03 - 1.06)# | 1.07 (1.01 - 1.12) | 1.04 (1.00 - 1.09) | 1.09 (1.02 - 1.17)# | 1.30 (1.15 - 1.48)# | 0.959 |
|  | Ever-smoker | 1.00 | 1.05 (1.03 - 1.08)# | 1.03 (0.96 - 1.10) | 1.04 (1.00 - 1.09) | 1.12 (1.05 - 1.20)# | 1.30 (1.15 - 1.47)# |  |
| GI_510 Diseases of esophagus | Never-smoker | 1.00 | 1.04 (0.92 - 1.17) | 1.15 (0.84 - 1.59) | 0.93 (0.71 - 1.24) | 1.08 (0.70 - 1.66) | 0.76 (0.24 - 2.36) | 0.977 |
|  | Ever-smoker | 1.00 | 1.01 (0.86 - 1.18) | 1.16 (0.81 - 1.65) | 0.91 (0.68 - 1.21) | 0.92 (0.59 - 1.42) | 1.20 (0.53 - 2.70) |  |
| GI_511 Gastro-esophageal reflux disease [GERD] | Never-smoker | 1.00 | 0.96 (0.85 - 1.10) | 1.24 (0.89 - 1.73) | 0.71 (0.50 - 1.00) | 0.84 (0.50 - 1.44) | 0.64 (0.16 - 2.56) | 0.442 |
|  | Ever-smoker | 1.00 | 0.85 (0.69 - 1.05) | 1.13 (0.70 - 1.83) | 0.93 (0.65 - 1.35) | 1.36 (0.83 - 2.21) | 1.21 (0.39 - 3.82) |  |
| GI_513 Peptic ulcer | Never-smoker | 1.00 | 1.03 (0.95 - 1.11) | 1.09 (0.89 - 1.34) | 1.01 (0.86 - 1.20) | 1.40 (1.12 - 1.75)# | 1.20 (0.73 - 1.97) | 0.182 |
|  | Ever-smoker | 1.00 | 0.96 (0.88 - 1.05) | 0.89 (0.70 - 1.13) | 1.09 (0.94 - 1.27) | 1.03 (0.83 - 1.29) | 1.05 (0.67 - 1.64) |  |
| GI_513.2 Gastric ulcer | Never-smoker | 1.00 | 1.00 (0.90 - 1.10) | 1.10 (0.85 - 1.44) | 1.06 (0.87 - 1.29) | 1.49 (1.15 - 1.93)# | 1.26 (0.71 - 2.23) | 0.690 |
|  | Ever-smoker | 1.00 | 0.96 (0.86 - 1.08) | 0.99 (0.74 - 1.32) | 1.01 (0.83 - 1.23) | 1.18 (0.91 - 1.52) | 0.83 (0.46 - 1.52) |  |
| GI_514 Gastrointestinal obstruction | Never-smoker | 1.00 | 1.18 (1.09 - 1.28)# | 0.96 (0.76 - 1.22) | 1.09 (0.92 - 1.31) | 1.21 (0.93 - 1.56) | 1.15 (0.65 - 2.04) | 0.168 |
|  | Ever-smoker | 1.00 | 1.19 (1.08 - 1.31)# | 1.34 (1.07 - 1.67) | 1.11 (0.94 - 1.31) | 1.05 (0.81 - 1.35) | 2.01 (1.36 - 2.96)# |  |
| GI_522 Gastrointestinal inflammation | Never-smoker | 1.00 | 1.01 (0.98 - 1.03) | 1.15 (1.07 - 1.24)# | 1.07 (1.01 - 1.14) | 1.11 (1.01 - 1.22) | 1.25 (1.05 - 1.49)# | 0.740 |
|  | Ever-smoker | 1.00 | 1.03 (0.99 - 1.07) | 1.13 (1.02 - 1.26) | 1.09 (1.02 - 1.17) | 1.19 (1.08 - 1.31)# | 1.12 (0.92 - 1.36) |  |
| GI_522.1 Inflammatory bowel disease | Never-smoker | 1.00 | 0.90 (0.70 - 1.17) | 1.05 (0.46 - 2.38) | 1.08 (0.57 - 2.05) | 0.89 (0.28 - 2.81) | 0.00 (0.00 - Inf) | 0.763 |
|  | Ever-smoker | 1.00 | 0.99 (0.70 - 1.39) | 1.04 (0.38 - 2.83) | 1.72 (0.97 - 3.05) | 1.11 (0.40 - 3.09) | 1.00 (0.14 - 7.35) |  |
| GI_522.8 Duodenitis | Never-smoker | 1.00 | 1.04 (0.71 - 1.54) | 1.27 (0.46 - 3.49) | 0.71 (0.26 - 1.94) | 0.49 (0.07 - 3.59) | 0.00 (0.00 - Inf) | 0.073 |
|  | Ever-smoker | 1.00 | 0.59 (0.34 - 1.04) | 0.00 (0.00 - Inf) | 0.43 (0.13 - 1.37) | 1.29 (0.39 - 4.25) | 0.00 (0.00 - Inf) |  |
| GI_522.9 Gastritis | Never-smoker | 1.00 | 1.01 (0.98 - 1.04) | 1.15 (1.07 - 1.25)# | 1.06 (1.00 - 1.13) | 1.12 (1.02 - 1.23) | 1.32 (1.11 - 1.58)# | 0.750 |
|  | Ever-smoker | 1.00 | 1.03 (0.99 - 1.07) | 1.14 (1.03 - 1.27) | 1.08 (1.01 - 1.17) | 1.19 (1.08 - 1.31)# | 1.15 (0.94 - 1.40) |  |
| GI_524 Functional intestinal disorder | Never-smoker | 1.00 | 0.91 (0.71 - 1.17) | 1.24 (0.76 - 2.03) | 0.72 (0.41 - 1.26) | 1.45 (0.73 - 2.85) | 2.14 (0.68 - 6.78) | 0.549 |
|  | Ever-smoker | 1.00 | 1.25 (0.87 - 1.79) | 0.95 (0.41 - 2.17) | 0.59 (0.27 - 1.29) | 1.57 (0.70 - 3.48) | 3.52 (1.08 - 11.45) |  |
| GI_526 Intestinal infection | Never-smoker | 1.00 | 1.02 (0.97 - 1.06) | 1.03 (0.92 - 1.17) | 1.02 (0.93 - 1.12) | 0.99 (0.86 - 1.14) | 0.99 (0.74 - 1.33) | 0.343 |
|  | Ever-smoker | 1.00 | 1.03 (0.96 - 1.10) | 1.13 (0.96 - 1.32) | 0.90 (0.80 - 1.01) | 1.10 (0.95 - 1.28) | 0.91 (0.65 - 1.28) |  |
| GI_527 Abdominal pain | Never-smoker | 1.00 | 1.10 (1.03 - 1.18)# | 1.08 (0.90 - 1.30) | 1.19 (1.05 - 1.35) | 1.32 (1.11 - 1.57)# | 1.72 (1.28 - 2.33)# | 0.623 |
|  | Ever-smoker | 1.00 | 1.02 (0.94 - 1.12) | 0.89 (0.70 - 1.14) | 1.24 (1.08 - 1.42)# | 1.28 (1.06 - 1.53) | 1.80 (1.33 - 2.43)# |  |
| GI_527.3 Functional dyspepsia | Never-smoker | 1.00 | 1.10 (1.03 - 1.18)# | 1.08 (0.90 - 1.30) | 1.19 (1.05 - 1.35) | 1.32 (1.11 - 1.57)# | 1.72 (1.28 - 2.33)# | 0.623 |
|  | Ever-smoker | 1.00 | 1.02 (0.94 - 1.12) | 0.89 (0.70 - 1.14) | 1.24 (1.08 - 1.42)# | 1.28 (1.06 - 1.53) | 1.80 (1.33 - 2.43)# |  |
| GI_535 Intestinal or peritoneal adhesions | Never-smoker | 1.00 | 1.44 (1.19 - 1.74)# | 0.66 (0.33 - 1.34) | 0.97 (0.63 - 1.49) | 0.89 (0.44 - 1.81) | 0.00 (0.00 - Inf) | 0.006 |
|  | Ever-smoker | 1.00 | 0.95 (0.71 - 1.26) | 1.73 (1.05 - 2.86) | 0.70 (0.41 - 1.20) | 1.18 (0.63 - 2.22) | 0.00 (0.00 - Inf) |  |
| GI_537 Abnormality of the peritoneum | Never-smoker | 1.00 | 1.08 (0.96 - 1.21) | 1.14 (0.84 - 1.54) | 0.81 (0.61 - 1.07) | 0.91 (0.60 - 1.39) | 0.96 (0.40 - 2.32) | 0.910 |
|  | Ever-smoker | 1.00 | 1.01 (0.87 - 1.18) | 0.93 (0.64 - 1.36) | 0.90 (0.69 - 1.18) | 1.04 (0.71 - 1.52) | 0.87 (0.36 - 2.10) |  |
| GI_537.1 Peritonitis | Never-smoker | 1.00 | 1.06 (0.95 - 1.19) | 1.17 (0.86 - 1.59) | 0.81 (0.62 - 1.08) | 0.89 (0.58 - 1.37) | 0.98 (0.41 - 2.38) | 0.949 |
|  | Ever-smoker | 1.00 | 1.00 (0.86 - 1.17) | 0.95 (0.65 - 1.40) | 0.87 (0.66 - 1.15) | 0.95 (0.63 - 1.41) | 0.87 (0.36 - 2.13) |  |
| GI_540 Hepatitis | Never-smoker | 1.00 | 1.31 (1.02 - 1.67) | 0.70 (0.26 - 1.88) | 0.55 (0.22 - 1.35) | 1.28 (0.47 - 3.50) | 1.50 (0.21 - 10.87) | 0.569 |
|  | Ever-smoker | 1.00 | 1.01 (0.76 - 1.35) | 0.35 (0.09 - 1.40) | 0.77 (0.39 - 1.51) | 1.86 (0.92 - 3.73) | 0.95 (0.13 - 6.85) |  |
| GI_540.1 Chronic hepatitis | Never-smoker | 1.00 | 1.29 (1.00 - 1.65) | 0.72 (0.27 - 1.95) | 0.58 (0.24 - 1.42) | 1.35 (0.50 - 3.69) | 1.59 (0.22 - 11.57) | 0.678 |
|  | Ever-smoker | 1.00 | 1.00 (0.72 - 1.38) | 0.23 (0.03 - 1.65) | 0.54 (0.22 - 1.34) | 1.68 (0.76 - 3.73) | 1.06 (0.15 - 7.73) |  |
| GI_542 Chronic liver disease | Never-smoker | 1.00 | 1.15 (1.07 - 1.23)# | 1.01 (0.82 - 1.25) | 0.93 (0.79 - 1.10) | 1.17 (0.91 - 1.49) | 1.88 (1.23 - 2.86)# | 0.427 |
|  | Ever-smoker | 1.00 | 1.10 (1.01 - 1.20) | 0.83 (0.63 - 1.08) | 1.04 (0.88 - 1.23) | 1.17 (0.92 - 1.49) | 2.20 (1.51 - 3.20)# |  |
| GI_542.1 Fibrosis and cirrhosis of liver | Never-smoker | 1.00 | 1.15 (1.04 - 1.28)# | 1.04 (0.75 - 1.44) | 0.89 (0.69 - 1.16) | 1.11 (0.76 - 1.63) | 1.83 (0.94 - 3.54) | 0.276 |
|  | Ever-smoker | 1.00 | 1.05 (0.93 - 1.19) | 0.76 (0.52 - 1.10) | 0.97 (0.77 - 1.23) | 1.19 (0.87 - 1.62) | 2.06 (1.25 - 3.42)# |  |
| GI_542.2 Fatty liver disease (FLD) | Never-smoker | 1.00 | 1.18 (1.06 - 1.30)# | 1.04 (0.76 - 1.43) | 0.93 (0.71 - 1.20) | 1.22 (0.84 - 1.78) | 2.30 (1.26 - 4.18)# | 0.856 |
|  | Ever-smoker | 1.00 | 1.17 (1.00 - 1.37) | 0.74 (0.43 - 1.26) | 0.98 (0.72 - 1.34) | 1.19 (0.75 - 1.88) | 1.62 (0.66 - 3.93) |  |
| GI_542.3 Hepatic failure | Never-smoker | 1.00 | 1.24 (0.95 - 1.64) | 0.75 (0.31 - 1.83) | 1.17 (0.65 - 2.12) | 0.89 (0.28 - 2.81) | 1.59 (0.22 - 11.55) | 0.577 |
|  | Ever-smoker | 1.00 | 0.95 (0.69 - 1.31) | 0.86 (0.35 - 2.12) | 1.63 (0.99 - 2.67) | 1.15 (0.46 - 2.85) | 5.51 (1.97 - 15.39)# |  |
| GI_542.7 Chronic nonalcoholic liver disease | Never-smoker | 1.00 | 1.14 (1.02 - 1.29) | 1.05 (0.73 - 1.51) | 0.87 (0.64 - 1.18) | 1.09 (0.69 - 1.71) | 1.45 (0.60 - 3.51) | 0.373 |
|  | Ever-smoker | 1.00 | 1.08 (0.93 - 1.25) | 0.62 (0.38 - 1.03) | 0.90 (0.67 - 1.21) | 0.97 (0.64 - 1.49) | 1.85 (0.95 - 3.62) |  |
| GI_550 Disorders of the gallbladder | Never-smoker | 1.00 | 1.08 (1.04 - 1.12)# | 1.01 (0.90 - 1.12) | 1.06 (0.98 - 1.15) | 1.25 (1.10 - 1.41)# | 1.59 (1.25 - 2.03)# | 0.865 |
|  | Ever-smoker | 1.00 | 1.09 (1.03 - 1.15)# | 0.97 (0.83 - 1.15) | 1.04 (0.94 - 1.17) | 1.07 (0.91 - 1.26) | 1.61 (1.22 - 2.13)# |  |
| GI_550.1 Gallstones [Cholelithiasis] | Never-smoker | 1.00 | 1.10 (1.05 - 1.16)# | 0.99 (0.86 - 1.13) | 0.98 (0.88 - 1.09) | 1.20 (1.02 - 1.41) | 1.57 (1.14 - 2.14)# | 0.549 |
|  | Ever-smoker | 1.00 | 1.16 (1.08 - 1.26)# | 1.02 (0.83 - 1.26) | 1.00 (0.86 - 1.16) | 0.99 (0.80 - 1.24) | 1.64 (1.13 - 2.40)# |  |
| GI_550.2 Cholecystitis | Never-smoker | 1.00 | 1.08 (1.02 - 1.14)# | 1.06 (0.89 - 1.25) | 1.18 (1.05 - 1.32) | 1.26 (1.05 - 1.50) | 1.80 (1.30 - 2.48)# | 0.858 |
|  | Ever-smoker | 1.00 | 1.06 (0.98 - 1.15) | 0.88 (0.68 - 1.14) | 1.10 (0.94 - 1.28) | 1.20 (0.97 - 1.48) | 1.51 (1.02 - 2.22) |  |
| GI_557 Gastrointestinal hemorrhage | Never-smoker | 1.00 | 1.06 (0.99 - 1.14) | 0.95 (0.78 - 1.14) | 1.07 (0.92 - 1.23) | 1.11 (0.89 - 1.39) | 1.25 (0.80 - 1.97) | 0.976 |
|  | Ever-smoker | 1.00 | 1.05 (0.97 - 1.13) | 0.95 (0.78 - 1.15) | 1.08 (0.95 - 1.23) | 1.16 (0.97 - 1.40) | 1.53 (1.10 - 2.13)# |  |

HRs and 95% CIs were calculated using stratified Cox regression models, stratified by age (in 5-year intervals), sex and living areas, adjusting for education, household income, occupation, marital status, alcohol consumption, passive smoking status, physical activity levels, primary cooking fuel, primary heating fuel usage, and consumption frequency of fresh fruits, fresh vegetables, meat, general obesity and abdominal obesity, as well as self-rated health status, prevalent hypertension and prevalent diabetes at baseline. *P* values for interactions of COPD with these factors were obtained from likelihood-ratio tests of the two models before and after adding the interaction term.

**P*<0.05; #*False discovery rate*<0.05*.*

Abbreviations: COPD, chronic obstructive pulmonary disease; HR, hazard ratio; CI, confidence interval

**Supplementary Figure 7. Associations of prevalent COPD at baseline with selected digestive diseases after excluding participants whose follow-up time was less than 2 years**

The centers of the squares represent the point estimates for HRs, and the edges represent the lower and upper bounds for 95% CIs. The sizes of the squares are in direct proportion to the reciprocals of the estimated standard errors of the effect sizes. HRs and 95% CIs were calculated using stratified Cox regression models, stratified by age (in 5-year intervals), sex and living areas, adjusting for education, household income, occupation, marital status, alcohol consumption, smoking status, passive smoking status, physical activity levels, primary cooking fuel, primary heating fuel usage, and consumption frequency of fresh fruits, fresh vegetables, meat, general obesity and abdominal obesity, as well as self-rated health status, prevalent hypertension and prevalent diabetes at baseline. **P*<0.05; #*False discovery rate*<0.05.

Abbreviations: COPD, chronic obstructive pulmonary disease; HR, hazard ratio; CI, confidence interval.

**Supplementary Figure 8. Associations of prevalent COPD at baseline with selected digestive diseases after additionally adjusting for anxiety**

The centers of the squares represent the point estimates for HRs, and the edges represent the lower and upper bounds for 95% CIs. The sizes of the squares are in direct proportion to the reciprocals of the estimated standard errors of the effect sizes. HRs and 95% CIs were calculated using stratified Cox regression models, stratified by age (in 5-year intervals), sex and living areas, adjusting for education, household income, occupation, marital status, alcohol consumption, smoking status, passive smoking status, physical activity levels, primary cooking fuel, primary heating fuel usage, and consumption frequency of fresh fruits, fresh vegetables, meat, general obesity and abdominal obesity, as well as self-rated health status, prevalent hypertension and prevalent diabetes, and anxiety at baseline.

**P*<0.05; #*False discovery rate*<0.05.

Abbreviations: COPD, chronic obstructive pulmonary disease; HR, hazard ratio; CI, confidence interval.

**Supplementary Figure 9. Associations of prevalent COPD at baseline with selected digestive diseases after excluding self-reported asthma at baseline**

The centers of the squares represent the point estimates for HRs, and the edges represent the lower and upper bounds for 95% CIs. The sizes of the squares are in direct proportion to the reciprocals of the estimated standard errors of the effect sizes. HRs and 95% CIs were calculated using stratified Cox regression models, stratified by age (in 5-year intervals), sex and living areas, adjusting for education, household income, occupation, marital status, alcohol consumption, smoking status, passive smoking status, physical activity levels, primary cooking fuel, primary heating fuel usage, and consumption frequency of fresh fruits, fresh vegetables, meat, general obesity and abdominal obesity, as well as self-rated health status, prevalent hypertension and prevalent diabetes at baseline. **P*<0.05; #*False discovery rate*<0.05.

Abbreviations: COPD, chronic obstructive pulmonary disease; HR, hazard ratio; CI, confidence interval.

**Supplementary Figure 10. Associations of prevalent COPD at baseline with selected digestive diseases after excluding participants who have reported the history of aspirin usage at baseline**

The centers of the squares represent the point estimates for HRs, and the edges represent the lower and upper bounds for 95% CIs. The sizes of the squares are in direct proportion to the reciprocals of the estimated standard errors of the effect sizes. HRs and 95% CIs were calculated using stratified Cox regression models, stratified by age (in 5-year intervals), sex and living areas, adjusting for education, household income, occupation, marital status, alcohol consumption, smoking status, passive smoking status, physical activity levels, primary cooking fuel, primary heating fuel usage, and consumption frequency of fresh fruits, fresh vegetables, meat, general obesity and abdominal obesity, as well as self-rated health status, prevalent hypertension and prevalent diabetes at baseline.

**P*<0.05; #*False discovery rate*<0.05.

Abbreviations: COPD, chronic obstructive pulmonary disease; HR, hazard ratio; CI, confidence interval.

**Supplementary Figure 11. Associations of prevalent COPD at baseline with selected digestive diseases without adjusting for BMI, abdominal obesity, prevalent hypertension, and prevalent diabetes**

The centers of the squares represent the point estimates for HRs, and the edges represent the lower and upper bounds for 95% CIs. The sizes of the squares are in direct proportion to the reciprocals of the estimated standard errors of the effect sizes. HRs and 95% CIs were calculated using stratified Cox regression models, stratified by age (in 5-year intervals), sex and living areas, adjusting for education, household income, occupation, marital status, alcohol consumption, smoking status, passive smoking status, physical activity levels, primary cooking fuel, primary heating fuel usage, and consumption frequency of fresh fruits, fresh vegetables, meat, as well as self-rated health status at baseline.

**P*<0.05; #*False discovery rate*<0.05.

Abbreviations: COPD, chronic obstructive pulmonary disease; HR, hazard ratio; CI, confidence interval.

**Supplementary Figure 12. Associations of prevalent COPD at baseline with selected digestive diseases in never-smokers without self-reported asthma and ever-smokers without self-reported asthma**

The centers of the squares represent the point estimates for HRs, and the edges represent the lower and upper bounds for 95% CIs. The sizes of the squares are directly proportional to the reciprocals of the estimated standard errors of the effect sizes. HRs and 95% CIs were calculated using stratified Cox regression models, stratified by age (in 5-year intervals), sex and living areas, adjusting for education, household income, occupation, marital status, alcohol consumption, passive smoking status, physical activity levels, primary cooking fuel, primary heating fuel usage, and consumption frequency of fresh fruits, fresh vegetables, meat, general obesity and abdominal obesity, as well as self-rated health status, prevalent hypertension and prevalent diabetes at baseline. *P* values for interactions of COPD with these factors were obtained from likelihood-ratio tests of the two models before and after adding the interaction term.

**P*<0.05; #*False discovery rate*<0.05*.*

Abbreviations: COPD, chronic obstructive pulmonary disease; HR, hazard ratio; CI, confidence interval.

**Supplementary Table 13. Sensitivity analysis accounting for competing risk of death for significant digestive outcomes**

| Diseases | SHR(95% CI) | P values |
| --- | --- | --- |
| Digestive diseases | 0.99(0.97-1.01) | 0.306 |
| Gastrointestinal inflammation | 1.05(1.01-1.08) | 0.004# |
| Gastritis | 1.05(1.01-1.08) | 0.007# |
| Abdominal pain | 1.14(1.07-1.22) | <0.001# |
| Functional dyspepsia | 1.14(1.07-1.22) | <0.001# |
| Disorders of the gallbladder | 1.00(0.95-1.05) | 0.930 |
| Cholecystitis | 1.08(1.01-1.16) | 0.025# |

SHRs and 95% CIs were calculated using stratified Fine-Gray competing risk models, treating deaths as competing events, stratified by age (in 5-year intervals), sex and living areas, adjusting for education, household income, occupation, marital status, alcohol consumption, smoking status, passive smoking status, physical activity levels, primary cooking fuel, primary heating fuel usage, and consumption frequency of fresh fruits, fresh vegetables, meat, general obesity and abdominal obesity, as well as self-rated health status, prevalent hypertension and prevalent diabetes at baseline.

#False discovery rate<0.05.

Abbreviations: COPD, chronic obstructive pulmonary disease; SHR, subdistribution hazard ratio; CI, confidence interval.

**Supplementary Table 14. Associations of genetically predicted COPD with selected digestive diseases**

| Diseases | No.event | HR(95% CI) | pval_1 | fdrp |
| --- | --- | --- | --- | --- |
| Digestive diseases | 18,062 | 1.98(1.37-2.86)# | 0.0003 | 0.004 |
| Gastrointestinal obstruction | 1,023 | 3.83(0.92-16.00) | 0.0652 | 0.1059 |
| Gastrointestinal inflammation | 7,778 | 2.08(1.19-3.64)# | 0.0105 | 0.0341 |
| Gastritis | 7,350 | 2.20(1.24-3.90)# | 0.0069 | 0.0299 |
| Abdominal pain | 1,474 | 4.50(1.26-16.05)# | 0.0204 | 0.0379 |
| Functional dyspepsia | 1,474 | 4.50(1.26-16.05)# | 0.0204 | 0.0379 |
| Chronic liver disease | 1,268 | 0.58(0.15-2.23) | 0.4268 | 0.5044 |
| Fibrosis and cirrhosis of liver | 567 | 0.84(0.11-6.70) | 0.8717 | 0.9443 |
| Fatty liver disease (FLD) | 479 | 0.17(0.02-1.47) | 0.1076 | 0.1554 |
| Disorders of the gallbladder | 4,014 | 2.82(1.33-5.97)# | 0.0068 | 0.0299 |
| Gallstones [Cholelithiasis] | 2,379 | 1.66(0.61-4.52) | 0.3191 | 0.4148 |
| Cholecystitis | 1,852 | 3.70(1.25-11.01)# | 0.0186 | 0.0379 |
| Gastrointestinal hemorrhage | 1,628 | 1.02(0.33-3.20) | 0.9693 | 0.9693 |

Genetically predicted probabilities of COPD were calculated using genetic IVs(GRSs) in logistic regression models adjusting for the top ten principal components and genetic array. The GRS used was ‘CKB GRS 5e8’ calculated from lead SNPs selected from the CKB-GWAS.. HRs and 95% CIs were computed using stratified Cox regression models, stratified by age (in 5-year intervals), sex and living areas, adjusting for education, household income, occupation, marital status, alcohol consumption, smoking status, passive smoking status, physical activity levels, primary cooking fuel, primary heating fuel usage, and consumption frequency of fresh fruits, fresh vegetables, meat, general obesity and abdominal obesity, self-rated health status, prevalent hypertension and prevalent diabetes at baseline, as well as the top ten principal components and genetic arrays.

*P<0.05; #False discovery rate<0.05.

Abbreviations: COPD, chronic obstructive pulmonary disease; HR, hazard ratio; CI, confidence interval; IV, instrumental variable; GRS, genetic risk score; CKB, China Kadoorie Biobank.

**Supplementary Figure 13. Associations of genetically predicted COPD with selected** **digestive diseases using IVs from the meta-GWAS**

The centers of the squares represent the point estimates for HRs, and the edges represent the lower and upper bounds for 95% CIs. The sizes of the squares are in direct proportion to the reciprocals of the estimated standard errors of the effect sizes. Genetically predicted probabilities of COPD were calculated using genetic IVs (GRSs) in logistic regression models adjusting for the top ten principal components and genetic array. The GRS used was ‘Meta GRS 5e8’ calculated from lead SNPs selected from the meta-GWAS of the CKB and BBJ populations. HRs and 95% CIs were computed using stratified Cox regression models, stratified by age (in 5-year intervals), sex and living areas, adjusting for education, household income, occupation, marital status, alcohol consumption, smoking status, passive smoking status, physical activity levels, primary cooking fuel, primary heating fuel usage, and consumption frequency of fresh fruits, fresh vegetables, meat, general obesity and abdominal obesity, self-rated health status, prevalent hypertension and prevalent diabetes at baseline, as well as the top ten principal components and genetic arrays.

**P*<0.05; #*False discovery rate*<0.05*.*

Abbreviations: COPD, chronic obstructive pulmonary disease; HR, hazard ratio; CI, confidence interval; IV, instrumental variable; GRS, genetic risk score; CKB, China Kadoorie Biobank. BBJ, Biobank Japan.

**Supplementary Figure 14. Associations of genetically predicted COPD with selected digestive diseases using IVs from the CKB-GWAS without adjusting for BMI, abdominal obesity, prevalent hypertension, and prevalent diabetes**

The centers of the squares represent the point estimates for HRs, and the edges represent the lower and upper bounds for 95% CIs. The sizes of the squares are in direct proportion to the reciprocals of the estimated standard errors of the effect sizes. Genetically predicted probabilities of COPD were calculated using genetic IVs(GRSs) in logistic regression models adjusting for the top ten principal components and genetic array. The GRS used was ‘CKB GRS 5e8’ calculated from lead SNPs selected from the CKB-GWAS. HRs and 95% CIs were computed using stratified Cox regression models, stratified by age (in 5-year intervals), sex and living areas, adjusting for education, household income, occupation, marital status, alcohol consumption, smoking status, passive smoking status, physical activity levels, primary cooking fuel, primary heating fuel usage, and consumption frequency of fresh fruits, fresh vegetables, meat, self-rated health status, as well as the top ten principal components and genetic arrays.

**P*<0.05; #*False discovery rate*<0.05*.*

Abbreviations: COPD, chronic obstructive pulmonary disease; HR, hazard ratio; CI, confidence interval; IV, instrumental variable; GRS, genetic risk score; CKB, China Kadoorie Biobank. BBJ, Biobank Japan.

**Supplementary Figure 15. Associations of genetically predicted COPD with selected digestive diseases using IVs from the BBJ-GWAS**

The centers of the squares represent the point estimates for HRs, and the edges represent the lower and upper bounds for 95% CIs. The sizes of the squares are in direct proportion to the reciprocals of the estimated standard errors of the effect sizes. Genetically predicted probabilities of COPD were calculated using genetic IVs(GRSs) in logistic regression models adjusting for the top ten principal components and genetic array. The GRS used was ‘BBJ GRS 5e8’ calculated from lead SNPs selected from the BBJ-GWAS. HRs and 95% CIs were computed using stratified Cox regression models, stratified by age (in 5-year intervals), sex and living areas, adjusting for education, household income, occupation, marital status, alcohol consumption, smoking status, passive smoking status, physical activity levels, primary cooking fuel, primary heating fuel usage, and consumption frequency of fresh fruits, fresh vegetables, meat, general obesity and abdominal obesity, self-rated health status, prevalent hypertension and prevalent diabetes at baseline, as well as the top ten principal components and genetic arrays.

**P*<0.05; #*False discovery rate*<0.05*.*

Abbreviations: COPD, chronic obstructive pulmonary disease; HR, hazard ratio; CI, confidence interval; IV, instrumental variable; GRS, genetic risk score; CKB, China Kadoorie Biobank. BBJ, Biobank Japan.

**Supplementary Figure 16. Associations of genetically predicted COPD with selected digestive diseases using IVs from the BBJ-GWAS after excluding two heterogeneous SNPs**

The centers of the squares represent the point estimates for HRs, and the edges represent the lower and upper bounds for 95% CIs. The sizes of the squares are in direct proportion to the reciprocals of the estimated standard errors of the effect sizes. Genetically predicted probabilities of COPD were calculated using genetic IVs(GRSs) in logistic regression models adjusting for the top ten principal components and genetic array. The GRS used was ‘BBJ GRS 5e8 nohet’ calculated from lead SNPs selected from the BBJ-GWAS, excluding two heterogeneous SNPs with opposite effects on COPD between the CKB and BBJ population. HRs and 95% CIs were computed using stratified Cox regression models, stratified by age (in 5-year intervals), sex and living areas, adjusting for education, household income, occupation, marital status, alcohol consumption, smoking status, passive smoking status, physical activity levels, primary cooking fuel, primary heating fuel usage, and consumption frequency of fresh fruits, fresh vegetables, meat, general obesity and abdominal obesity, self-rated health status, prevalent hypertension and prevalent diabetes at baseline, as well as the top ten principal components and genetic arrays.

**P*<0.05; #*False discovery rate*<0.05*.*

Abbreviations: COPD, chronic obstructive pulmonary disease; HR, hazard ratio; CI, confidence interval; IV, instrumental variable; GRS, genetic risk score; CKB, China Kadoorie Biobank. BBJ, Biobank Japan.
